# The failure of microglia to digest developmental apoptotic cells contributes to the pathology of RNASET2‐deficient leukoencephalopathy

**DOI:** 10.1002/glia.23829

**Published:** 2020-03-25

**Authors:** Noémie Hamilton, Holly A. Rutherford, Jessica J. Petts, Hannah M. Isles, Thomas Weber, Marco Henneke, Jutta Gärtner, Mark J. Dunning, Stephen A. Renshaw

**Affiliations:** ^1^ The Bateson Centre, Department of Infection Immunity and Cadiovascular Disease, University of Sheffield Sheffield UK; ^2^ Department of Pediatrics and Adolescent Medicine, Division of Pediatric Neurology University Medical Center Göttingen Göttingen Germany; ^3^ The Bioinformatics Core Sheffield Institute of Translational Neuroscience, University of Sheffield Sheffield UK

**Keywords:** developmental apoptosis, leukodystrophy, lysosomal storage disorder, microglia, microglia depletion, RNaseT2, zebrafish

## Abstract

The contribution of microglia in neurological disorders is emerging as a leading disease driver rather than a consequence of pathology. RNAseT2‐deficient leukoencephalopathy is a severe childhood white matter disorder affecting patients in their first year of life and mimicking a cytomegalovirus brain infection. The early onset and resemblance of the symptoms to a viral infection suggest an inflammatory and embryonic origin of the pathology. There are no treatments available for this disease as our understanding of the cellular drivers of the pathology are still unknown. In this study, using a zebrafish mutant for the orthologous *rnaset2* gene, we have identified an inflammatory signature in early development and an antiviral immune response in mature adult brains. Using the optical transparency and the ex utero development of the zebrafish larvae we studied immune cell behavior during brain development and identified abnormal microglia as an early marker of pathology. Live imaging and electron microscopy identified that mutant microglia displayed an engorged morphology and were filled with undigested apoptotic cells and undigested substrate. Using microglia‐specific depletion and rescue experiments, we identified microglia as drivers of this embryonic phenotype and potential key cellular player in the pathology of RNAseT2‐deficient leukoencephalopathy. Our zebrafish model also presented with reduced survival and locomotor defects, therefore recapitulating many aspects of the human disease. Our study therefore placed our *rnaset2* mutant at the forefront of leukodystrophy preclinical models and highlighted tissue‐specific approaches as future therapeutic avenues.

## Main Points

- Loss of *rnaset2* results in inflammation and lysosomal‐deficient microglia failing to digest dying neurons.
- Targeting microglia restores embryonic microglial defects to clear neurodevelopmental apoptosis.
- Mutants have reduced survival and locomotor defects.

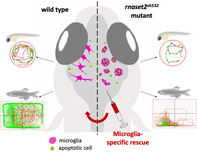

## 1 INTRODUCTION

In the central nervous system (CNS), developmental apoptosis is critical for the formation of a healthy brain, eliminating excess neurons produced during development (Dekkers, Nikoletopoulou, & Barde, 2013; Meier, Finch, & Evan, 2000; Nijhawan, Honarpour, & Wang, 2000). Dying neurons are cleared by the main phagocyte in the CNS—the microglia—as part of a necessary immune response which allows natural clearing of apoptotic neurons. However, disordered immunity in the brain is also a feature of many neurological disorders and plays a crucial role in the onset and progression of many neurodegenerative and white matter diseases.

White matter disorders such as leukodystrophies are characterized by myelin defects and white matter degeneration. The early onset of clinical symptoms includes loss of motor function and cognitive decline, with patients often dying at a young age (Bielschowsky & Henneberg, 1928). Patients as young as a few months old can present with neurological deficits, suggesting a fetal origin of the disease. At least 200 rare leukodystrophies have been identified to date, many of which are caused by the dysfunction of oligodendrocytes and subsequent loss of myelin integrity (United Leukodystrophy Foundation, 2019). More recently, the contribution of microglia and neuroinflammation to disease pathology has been recognized, prompting reclassification of these diseases and a requirement for further understanding of the mechanisms underlying disease progression (Kevelam et al., 2016; van der Knaap & Bugiani, 2017).

Microglial activation and increased immune activity have recently been detected even before the onset of myelin loss. Evidence from postmortem studies suggested that microglial activation preceded the reduction of myelin viability in patients suffering from metachromatic leukodystrophy and X‐linked adrenoleukodystrophy (Bergner et al., 2019). Mouse models of Krabbe's disease, another fatal leukodystrophy, showed similar microglial activation before myelin loss, with elevated innate immune markers even before microglial activation (Potter et al., 2013; Snook et al., 2014). The phenotype of another common leukodystrophy, Aicardi Goutières Syndrome (AGS), resembled a cytomegalovirus brain infection with early upregulation of type I interferon being central to the pathology (Crow & Manel, 2015). Understanding whether the immune system is a driver of the disease rather a consequence may uncover new avenues for therapeutic interventions to treat these otherwise incurable diseases.

The development of animal models to study leukodystrophies in vivo has been invaluable for testing therapies (Biffi et al., 2004; Launay et al., 2017; Marshall et al., 2018; Priller et al., 2001); however, many of those models fail to recapitulate the key aspects of the human disease. We have previously generated a zebrafish model for the Ribonuclease T2 (RNAseT2)‐deficient leukoencephalopathy through knockout of the zebrafish *rnaset2* gene, orthologous to its human equivalent (Haud et al., 2011). Similar to AGS, RNAseT2‐deficient leukoencephalopathy is another severe leukodystrophy with clinical manifestations resembling a congenital cytomegalovirus brain infection. This disorder affects children in their first year of life and is characterized by white matter lesions, subcortical cysts and calcification, with severe psychomotor and sensorineural hearing impairments (Henneke et al., 2009). We and others have shown that loss of RNAseT2 causes a lysosomal storage disorder (LSD) resulting in the accumulation of ribosomal RNA (rRNA) in lysosomes due to defects in ribosome autophagy (ribophagy) (Haud et al., 2011; Hillwig et al., 2011). Adult *rnaset2‐*deficient zebrafish develop white matter lesions in adult brains, recapitulating this aspect of the human pathology. Furthermore, *rnaset2* deficiency triggers increased expression of neuroinflammatory markers in the brain, further supporting a role for immune activation in driving pathogenesis in leukodystrophies (Haud et al., 2011). However, as for most rare monogenic leukodystrophies, there is no treatment and the exact contribution of the immune system to the pathology is still obscure.

We hypothesized that the early onset of RNAseT2‐deficient leukoencephalopathy is caused by abnormal microglial activity during neurodevelopment. In this current study, we utilize the ex utero development of zebrafish larvae and its optical transparency to study the impact of loss of *rnaset2* early during brain development. Gene expression analysis identified an upregulation of the immune system in brains of larval and adult mutant. Using confocal imaging and high‐resolution electron microscopy (EM), we identified profoundly abnormal microglia in *rnaset2‐*deficient larvae, displaying an engorged morphology and filled with undigested apoptotic cells. Using microglia depletion, we showed that the increased number of apoptotic cells is not due to increased neuronal cell death but to microglial failure at digesting apoptotic bodies. Tissue‐specific expression of *rnaset2* targeting neurons and microglia rescued the embryonic pathological phenotype in *rnaset2* zebrafish mutants. Additionally, we found that loss of *rnaset2* was associated with reduced survival, and locomotor defects at larval and adult stages.

Our findings identified dysfunctional microglia as early markers of the pathology, highlighting a critical role for microglia in disease development from its early stages. We propose a new mechanism by which undigested storage material, previously identified only in neurons, accumulates in microglia during neurodevelopmental apoptosis. This bottleneck phenomenon identifies the microglia as the point of congestion and final compartment for the undigested storage material that might cause the identified neuroinflammation in the early brain, with negative impact on brain integrity. Moreover, our results highlight the benefit of neuron and microglia specific treatment approaches, opening the possibility of gene therapy and bone marrow transplant to treat this rare leukodystrophy.

## 2 METHODS

### 2.1 Zebrafish husbandry and ethics

All zebrafish were raised in the Bateson Centre at the University of Sheffield in UK Home Office approved aquaria and maintained following standard protocols (Nüsslein‐Volhard & Dham, 2002). Tanks were maintained at 28°C with a continuous recirculating water supply and a daily light/dark cycle of 14/10 hr. All procedures were performed on embryos less than 5.2 dpf which were therefore outside of the Animals (Scientific Procedures) Act, to standards set by the UK Home Office. We used the *Tg*(*mpeg1*:*mCherryCAAX*)*sh378* labeling the membrane of macrophages and microglia (Bojarczuk et al., 2016), the previously ENU‐generated *rnaset2* ^*AO127*^ mutant and *nacre* wild type (WT) to generate the new CRISPR/Cas9 *rnaset2* ^*sh532*^ mutant.

### 2.2 Microarray experiment and analysis

RNA was isolated from pooled dechorionated embryos at 28 hr postfertilization (hpf), 3 day postfertilization (dpf) and dissected whole adult brains using TRIzol (Invitrogen), following the manufacturer's protocol. RNA integrity was confirmed using the Agilent 2100 BioAnalyzer, using only samples with an RNA integrity number of at least 8.

Genome‐wide expression profiling was performed using the Zebrafish (v3) Gene Expression 4x44K Microarray (Agilent GPL14664) containing 43,803 probes according to the manufacturer's instructions. The probe sequences for this array platform were reannotated using the ReAnnotator pipeline (Arloth, Bader, Röh, & Altmann, 2015) against reference genome danRer11 in order to obtain updated gene symbols.

Scanned raw data were normalized using quantile normalization, and then filtered using the ReAnnotator results so that only probes matching the exons of their target gene were retained. In the case of genes with more than one probe, the probe with the highest inter‐quartile range was chosen as the representative probe for the gene.

Differential expression analysis was conducted using a linear model framework and empirical Bayes' shrinkage implemented in the limma Bioconductor package (Ritchie et al., 2015). Genes with an adjusted *p*‐value <.05 were taken forward to enrichment analysis with the clusterProfiler Bioconductor package (Yu, Wang, Han, & He, 2012). Accession number for deposited dataset: GSE138493.

### 2.3 Generation of *rnaset2* ^*sh532*^ mutant

Synthetic SygRNA consisting of gene specific CRISPR RNAs (crRNA) (Sigma) and transactivating RNAs (tracrRNA) (Merck) in combination with cas9 nuclease protein (Merck) was used for gene editing. TracrRNA and crRNA were resuspended to a concentration of 50 μM in nuclease free water containing 10 mM Tris–HCl pH8. SygRNA complexes were assembled on ice immediately before injection using a 1:1:1 ratio of crRNA:tracrRNA:Cas9 protein. We used CHOPCHOP to design the following crRNA sequences, where the PAM site is indicated in brackets: *rnaset2* (CCG)AGATCTGCTAGAACCATCTT. A 2 nl drop of SygRNA:Cas9 protein complex was injected into one‐cell stage embryos. The resulting *rnaset2* crispants were raised and screened (see below) to select a suitable mutation for a stable line.

To determine the efficiency of CRISPR/Cas9 in inducing site‐specific mutations in injected larvae, we used high‐resolution melt curve (HMRC) analysis or PCR followed by *Mwo1* restriction digest (Sup. Figure 4C,D). Genomic DNA (gDNA) was extracted from individual larvae at 2dpf. Larvae were placed individually in 90 μl 50 mM NaOH and boiled at 95° for 20 min. A total of 10 μl Tris–HCL pH8 was added to adjust the pH and mixed thoroughly. Gene specific primers were designed using the Primer 3 web tool (http://primer3.ut.ee/) and primers with the following sequences were used to amplify a 142 bp region: HRMCrnaset2_fw ACATACTACCAGAAATGGAG, HMRCrnaset2_rev GTAGTGCCTAAATGCATTTG. HMRC analysis was run using the CFX96 Bio‐Rad quantitative PCR (qPCR) machine with the Bio‐Rad Precision Melt Analysis software using the following reaction: gDNA 1 μl, 5μl DyNAmo Flash SYBR Green (Thermo Fisher), 0.5 μl of each primers 10 μM and 4 μl of water. The program used was: Step 1:95°C for 2 min, Step 2:95°C for 10 s, Step 3:60°C for 30 s, Step 4:72°C for 30 s, Step 5:95°C for 30 s, Step 6:60°C for 10 min, Step 7:95°C for 20 s with Steps 2–4 repeated 44 times and increment of 0.2°C every 10 s between Steps 6 and 7.

Potential founders were outcrossed and gDNA was extracted from eight embryos from each progeny. HMRC was used to identify INDELS in progenies, which were subsequently raised. Individual F1 adults were fin clipped and an 8 bp deletion causing a frame shift and an early stop codon in a similar region as our previous *rnaset2* mutant was kept, given the “*sh532*” code and outcrossed to the *Tg*(*mpeg1*:*mCherryCAAX*)*sh378* line. Heterozygous animals were in‐crossed and progeny genotyped. Homozygous (*rnaset2* ^*sh532*^) and wild type siblings (WT) were selected, separated, and raised to adulthood. All experiments were done on in‐crosses from each tank, except when stated otherwise in Sup. Figure S7.

### 2.4 Quantitative PCR

RNA was extracted from brains of 3‐month‐old zebrafish suing the Trizol/chloroform method. cDNA was synthetized using the SuperScript II kit (Invitrogen) with 2 μg of RNA following manufacturer instructions and diluted in 1:20 for qPCR. qPCR primers (Supplementary Table S1) were tested for efficiency (85–105%) using a cDNA serial dilution. The qPCR reaction was run in a CFX96 Bio‐Rad machine as follow: 2 μl of cDNA, 5 μl DyNAmo Flash SYBR Green (Thermo Fisher), 0.5 μl of each primers 10 μM, and 3 μl of water. The program used was: Step 1:95°C for 2 min, Step 2:95°C for 10 s, Step 3:60°C for 30 s, Step 4:72°C for 25 s, Step 5:95°C for 30 s, Step 6:65°C for 10 s, Step 7:95°C for 20 s with Steps 2–4 repeated 39 times and increment of 0.2°C every 10 s between Steps 6 and 7. ΔCT was calculated using combined *rpl13* and *ef1α* as reference genes and expression relative to endogenous control was calculated using the 2^(−ΔCT).

### 2.5 Acridine orange assay

A pool of 20 5dpf WT and *rnaset2* ^*sh532*^ mutant zebrafish larvae were incubated for 20 min in 5 μg/ml of acridine orange (Life Technologies) diluted in zebrafish E3 medium in the dark. After three 10 min washed in clean E3, larvae were sedated and chopped finely with a scalpel then transferred to a tube containing PBS with Liberase (Roche, reference 05401020001) at 40 μg/ml. Samples were incubated at 37°C for 30 min, with triturating of the mixture every 10 min to dissociate into a single cell suspension. Samples were centrifuged at 1,000*g* for 6 min and resuspended in Leibovitz 15 media containing 20% FBS and 5 mM EDTA. After filtration to remove clumps of cells, samples were analyzed using an ATTUNE Flow cytometry machine using the blue laser.

### 2.6 Microglia morphology and count

To assess microglia morphology, 5dpf WT and *rnaset2* ^*sh532*^ mutant zebrafish larvae in the *Tg(mpeg1*:*mCherryCAAX)*sh378 background were anesthetized and embedded in low melting point agarose containing tricaine (0.168 mg/ml; Sigma‐Aldrich) and imaged using a 40× objective on a UltraVIEW VoX spinning disk confocal microscope (PerkinElmer Life and Analytical Sciences) confocal spinning disk microscope. A total of 100 μm stacks were acquired using 0.5 μm slices. For image analysis, stacks of 50 μm were used to create a maximum projection and contours of each microglia (avoiding pigment cells) were drawn using a pen tablet (Intuos from Wacom). Using Fiji, we measured the circularity index (0–1) of each microglia and used these values to assess the circularity of each cell with the value of 0 being not circular and 1 as being perfectly circular.

The same images were used to quantify the number of vacuoles in each microglia. We used the labeled microglia membrane from *Tg*(*mpeg1*:*mCherryCAAX*)sh378 to manually count vacuoles. A number of vacuoles in microglia exceeding 20 were capped due to the difficulty to be accurate after this number.

For microglia count, 5dpf larvae were imaged under the 10× objective of the spinning disk microscope. Stacks of 100 μm were generated with 2 μm thickness and microglia were counted in the optic tectum region of each larvae by drawing a region of interest around the optic tectum first using the reference bright‐field image.

### 2.7 Generation of *irf8* morphants and crispants

The *irf8* modified antisense oligonucleotide‐morpholino (Gene Tools) was used as previously reported by injecting 1 nl of 0.5 mM irf8 morpholino in the yolk of one‐cell stage embryos (Li, Jin, Xu, Shi, & Wen, 2011).

For crispant experiments, preparation and injection proceeded a described above for the generation of *rnaset2* mutant. The *irf8* crRNA sequence was kindly shared by Dr Daniel Lysko, Dr Will Talbot: *irf8* GCGGTCGCAGACTGAAACAG(TGG) and 2 nl was injected at 50 ng/μl. Successful *irf8* crispant were phenotypically identified by injecting the SygRNA:Cas9 complex into the *Tg*(*mpeg1*:*mCherryCAAX*)*sh378* and selecting embryos with no microglia at 5dpf. For control injection, we replaced *irf8* crRNA by scrambled crRNA: GACCTGAGGGAGCAAGATCC(TGG).

### 2.8 TUNEL and 4C4 antibody dual staining

Fish larvae at 5dpf were fixed in 4% PFA and the TUNEL assay was performed according to standard protocol using ApopTag Kit (Millipore). Briefly, 5dpf larvae were incubated with proteinase K (20 μg/ml) for 2 hr to permeabilize the tissue. Samples were fixed for 20 min in PFA at room temperature before being placed at −20°C in 1:2 acetone: ethanol for 7 min. Following incubation at 37°C with 50 μl equilibration buffer for 1 hr, the reaction solution (16 μl TdT enzyme and 30 μl reaction buffer; Apoptag Kit) was added to the embryos—again, incubating at 37°C for 90 min. Embryos were then placed in 200 μl stop buffer (Apoptag kit) for 2 hr at 37°C, before placing in antibody and blocking solution (62 μl anti‐Dig Fluorescein and 68 μl blocking solution) overnight at 4°C. The following morning, samples were thoroughly washed in PBST (PST + 0.1% Tween) and fixed in PFA for 30 min at room temperature. All liquid was removed and embryos thoroughly rinsed with PBST between each stage (5 min washes at room temperature, repeated three to four times as needed). Samples at this stage can be imaged for TUNEL and costained with antibodies by following a basic immunofluorescent staining protocol. In brief, samples were transferred to water and cracked with cold acetone at −20°C for 20 min. After water and then PBST rinses, samples were blocked at RT in 10% sheep serum, 0.8% TritonX‐100, 1%BSA in PBST for 3 hr then incubated for 4 days in 1:50 4C4 antibody. After multiples PBST rinses, samples were incubated in secondary Cy5 anti‐mouse antibody at 1:500 for 2 days. Samples were then rinses in PBST and mounted in low melting point agarose for imaging.

Samples were imaged on the inverted UltraVIEW VoX spinning disk confocal microscope (PerkinElmer Life and Analytical Sciences) using the bright‐field and GFP channel by acquiring stacks of approximately 100 μm with 2 μm per slice. Using Fiji, the optic tectum region was outlined with the free hand drawing tool using the bright‐field image and counting of number of apoptotic cells was performed using the automatic thresholding and particle count. Manual colocalization analysis was performed between TUNEL and 4C4. Analysis was blinded until after counting was performed.

### 2.9 Electron microscopy

Three WT and *rnaset2* ^*sh532*^ mutant larvae at 5dpf were fixed in 2.5% glutaraldehyde/0.1 M sodium cacodylate overnight and postfixed 2% aqueous osmium tetroxide. Samples were dehydrated through a graded series of ethanol, and cleared in epoxypropane overnight on a rotor and then infiltrated in 50/50 Araldite resin. This mixture was replaced with two changes over 8 hr of fresh Araldite resin before being embedded and cured in a 60°C oven for 48–72 hr. Ultrathin sections, approximately 85 nm thick, were cut on a Leica UC6 ultramicrotome onto 200 mesh copper grids. These were stained for 10 min with saturated aqueous Uranyl Acetate followed by Reynold's lead citrate for 5 min. Sections were examined using an FEI Tecnai Transmission Electron Microscope at an accelerating voltage of 80 kv. Electron micrographs were recorded using Gatan Orius 1000 digital camera and Gatan Digital Micrograph software.

### 2.10 Apoptotic cells phagocytosis assays

Mutant and WT in the *Tg*(*mpeg1*:*mCherryCAAX*)*sh378* background were injected with equal ratio of phenol red solution, pDest (*ubiq*:*secAnnexinV‐mVenus*) construct (Morsch et al., 2015) at 80 ng/μl and tol2 mRNA at 150 ng/μl at one‐cell stage. The construct also contained a red eye marker to identify positively injected fish. For time‐lapse analysis, positive 3dpf fish were sedated in 4.2% Tricaine and one mutant and one WT were embedded together in low melting point agarose into the capillary of the light sheet fluorescence microscope. Time‐lapse was performed on both samples at the same time, acquiring images every 3 min for 2 hr. This was repeated twice. To quantify the number of microglia containing a mVenus positive cells, multiple fish were embedded into a dish containing a coverslip and imaged on the inverted UltraVIEW VoX spinning disk confocal microscope (PerkinElmer Life and Analytical Sciences), acquiring images every 3 min for 2 hr. Uptake of apoptotic cells was quantified throughout the video by all microglia and final number were used for quantification. This experiment was repeated three times on different batches of larvae.

### 2.11 Cloning of rescue constructs and generation of F1 lines

The *rnaset2* cDNA was amplified from the PCGlobin vector previously used (Haud et al., 2011) using the following *Attb* primers: rnaset2_AttB1fwd GGGGACAAGTTTGTACAAAAAAGCAGG‐CTGGATGAGATTCATTGCATTTGCTG, rnaset2_AttB2rev GGGGACCACTTTGTACAAGAA‐AGCTGGGTGCTACGCTTGCACCGGTGGGTA. Using the BP reaction, the PCR product was cloned into the p221DONOR vector which was used for the following LR reactions. To create the positive control construct *ubi*:*rnaset2*:*pA* construct, we used the p5′E‐*ubi* (Mosimann et al., 2011), pME‐*rnaset2*, p3′E‐polyA into the *cryCFPpDest* vector. To create the *mpeg1*:*rnaset2*:*pA* construct, we used the p5′E‐*mpeg1* (Ellett, Pase, Hayman, Andrianopoulos, & Lieschke, 2011), pME‐*rnaset2*, p3′E‐polyA into the *cryCFPpDest* vector. To create the *huc*:*rnaset2*:*pA* construct, we used the p5′E‐*huc* (cloned into p5′E using the following primers and restriction sites: Xhohuc_fwd CGACTGCTCGAGCTTCCGGCTCGTATGTTGTG and Bamhuc_rev GCAGGATCCGGTCCTTCGATTTGCAGGTC), pME‐*rnaset2*, p3′E‐polyA into the *cryCFPpDest* vector. Injections in *rnaset2* ^*sh532*^ mutant consisted of equal ratio of phenol red solution, vectors at 200 ng/μl and tol2 mRNA at 150 ng/μl at one‐cell stage. Blue‐eyed larvae were selected at 5dpf before imaging and fixing for F0 analysis. For F1 analysis, F0 injected larvae were raised and founders were identified by screening their progeny for blue‐eyed larvae. These larvae were fixed at 5dpf for TUNEL staining and immunofluorescence staining.

### 2.12 Adult behavior tests

Daily observations of adult fish ensured early detection of tilted swimmers. These fish were closely monitored until they started spiraling at which point they were humanly culled. Scoring of animals with spiraling swimming phenotype was added up to have a monthly total. WT adult fish did not develop tilted or spiraling swimming at any stage. Survival was plotted onto a survival curve in GraphPad Prism and a Log Rank Test with Mantel Cox posttest was used to determine statistical difference between the two survival curves.

Behavioral analysis on nontilted swimmers was performed using the Zebrabox tracking system and Zebralab software (ViewPoint Life Science, France). Eight‐month‐old WT and mutant fish were matched for sex and size, before being placed individually into open field tanks and allowed to habituate for 30 min before recording. The walls of each tank were covered with white paper to ensure the animals were unable to interact or be distracted by their surroundings. Movement was tracked over a period of 10 min under constant light conditions. For quadrant analysis, the Zebrabox software was programmed such that the open field tank was divided into four equal quadrants, where Quadrant 1 was the most active quadrant while Quadrant 4 was the least. Distance traveled by each fish was pooled and averaged—both in total, and within each quadrant—in Microsoft Excel, with the resulting data analyzed in GraphPad Prism.

### 2.13 Larval behavior light/dark behavior test

As above, behavioral analysis was performed using the Zebrabox tracking system and Zebralab software (ViewPoint Life Science).

To assess swimming behavior in response to light, 5dpf embryos were transferred into 48‐well plates (one embryo per well) and allowed to habituate overnight. Swimming distance during a 1‐min interval was recorded for 20 min during an alternating dark–light protocol (5 min dark, followed by 5 min light). Total distance was calculated as the sum of the distance swam across all 20 intervals for a single fish. Response to changes in light intensity was defined as the distance swam in the minute after each light‐to‐dark or dark‐to‐light transition. Distance traveled by each fish was pooled and averaged and the resulting data were analyzed using GraphPad Prism. Outliers that had not been tracked accurately over the recording period were manually excluded based on abnormal angles and straight lines generated by the Zebralab software.

### 2.14 Statistical analysis

All statistical analysis were performed in GraphPad Prism where data was entered using either a column (two samples, one variable only) or a grouped table (more than two samples or two variables). Sample distribution was assessed using frequency of distribution analysis. Nonparametric tests were used for not normally distributed dataset with posttest (for multiple comparisons) described for each experiment. All experiments were repeated three times using different batches of larvae born on different dates, with the number of biological replicates and n (experimental unit) number stated for each experiment in figure legends. Contingency tables were used for adult zebrafish behavior analysis with Fisher's exact test.

*p*‐Values are indicated and a star system is used instead for graph with multiple comparisons: * = *p* < .05, ** = *p* < .01, *** = *p* < .001, **** = *p* < .0001. Following the recommendation of the American Statistical Association, we do not associate a specific *p* value with significance (Wasserstein, Schirm, & Lazar, 2019).

## 3 RESULTS

### 3.1 Loss of *rnaset2* triggers an inflammatory response in the brain

**Figure 1:**
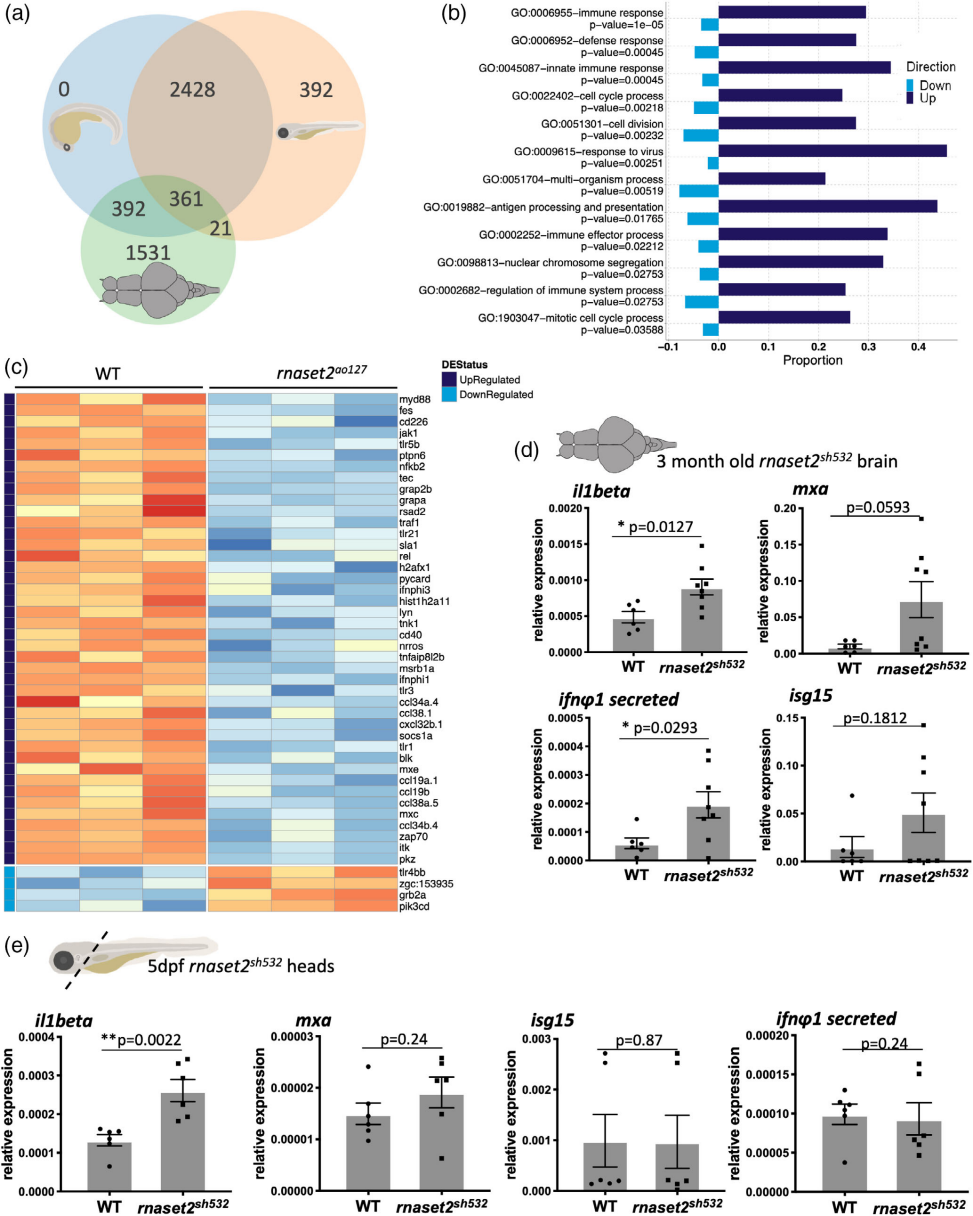
Loss of *rnaset2* triggers an inflammatory response in the brain. (a) Venn diagram of differentially expressed genes across the three timepoints of *rnaset2* ^*AO127*^ mutant and wild type (WT) samples used for microarray experiment. (b) Differential expression (DE) graph showing the enriched pathways identified by clusterProfiler in adult brain. The proportion of individual genes belonging to each pathway is displayed in dark blue for upregulated and light blue for downregulated expression. The *p*‐value for the enrichment of each pathway is stated below the pathway name. (C) Heatmap of genes belonging to innate immune response GO pathway showing normalized expression values and all replicates of the adult timepoint. Genes are ordered according to the magnitude of difference between *rnaset2* ^*AO127*^ mutant and WT samples (with red indicating higher expression in mutant). Each row is annotated with the gene name and color‐coded bars at the top to indicate whether the gene is upregulated (dark blue) or downregulated (light blue) regulated, or not significant at the adjusted *p*‐value threshold of .05. The −10log of the adjusted *p*‐value is also shown. (d,e) Quantitative PCR (qPCR) analysis of inflammatory *il1beta* and antiviral response genes: *mxa*, *isg15*, *ifnΦ1* from dissected brain of 3‐month‐old (d) and dissected heads of 5dpf (e) WT siblings and *rnaset2* ^*sh532*^. Expression relative to two reference genes combined *rpl13* and *ef1α*. *n* = 8 (d) and *n* = 6 (e), two‐tailed Mann–Whitney *U* test, *p* value shown on individual graphs [Color figure can be viewed at wileyonlinelibrary.com]

Human patients suffering from the loss of functioning RNASET2 present with clinical manifestations resembling a cytomegalovirus infection and therefore suggesting an important role for the immune system in the pathology of the disease. To investigate changes in the immune response, we performed transcriptomics analysis at different timepoints using the previously published zebrafish model for this disease, the *rnaset2* ^*AO127*^ mutant (Haud et al., 2011). Microarray analysis was performed on whole *rnaset2* ^*AO12*^ and WT siblings during early development at 28 hr postfertilization (hpf) and 3 days postfertilization (dpf), and on dissected brains of 1‐year‐old adult zebrafish (Figure 1a). Biological pathway analysis identified 12 pathways differentially regulated in adult brain, including seven involved in the immune system (Figure 1b). Genes from “immune system,” “defense response,” and “innate immune response” pathways were mostly upregulated in mutant samples (Figure 1b). Focusing specifically on the innate immune response, 35% of the genes were upregulated (Figure 1c) suggesting a strong immune response in the brain of adult mutants. Biological pathway analysis did not reveal significant changes in 28hpf samples, and only identified variations in cell cycle pathways at 3dpf (Sup. Figure S1). Additionally, our primary analysis showed that biological repeats from each time point clustered together with the adult brain samples showing a clear separation from the embryonic time points (Sup. Figure S2).

Interestingly, most genes in the GO “response to virus” pathway were upregulated in brains of adult *rnaset2* ^*AO127*^ deficient zebrafish (Sup. Figure S3), thus suggesting that the mutant zebrafish also present with a “viral‐infection‐like” phenotype. This further demonstrated that our *rnaset2* mutant recapitulated the human disease resembling a cytomegalovirus brain infection (Henneke et al., 2009).

### 3.2 CRISPR/Cas9 *rnaset2* ^*sh532*^ mutant recapitulates previous *rnaset2* ^*AO127*^ mutant

To further characterize the immune response triggered with the loss of *rnaset2*, we generated a second mutant allele by CRISPR/Cas9 in the *rnaset2* gene by targeting the same exon as our previous *rnaset2* ^*AO127*^ mutant, as our previous mutant line was lost. This new *rnaset2* ^*sh532*^ mutant allele possesses an 8 bp deletion in Exon 5 (Sup. Figure S4a), creating a premature STOP codon before the second catalytic domain and resulting in a truncated rnaset2 protein (Sup. Figure S4b), similar to our previous mutant (Haud et al., 2011). Homozygous and WT sibling pairs were raised from a heterozygous in‐cross and genotyped by HRMC analysis and/or restriction digest (Sup. Figure S4c,d). These fish were used to generate F2 homozygous (*rnaset2* ^*sh532*^ *)* and WT siblings which were viable and fertile, as observed with our previous mutant. The previous *rnaset2* ^*AO127*^ mutant had a characteristic high uptake of acridine orange, a dye accumulating in lysosomes highlighting the lysosomal storage defects (Haud et al., 2011), a phenotype which is recapitulated by our *rnaset2* ^*sh532*^ mutant (Sup. Figure S5).

To determine whether immune response genes were also upregulated in the CRISPR mutant, we performed qPCR analysis on brains from 3‐month‐old *rnaset2* ^*sh532*^ mutant and WT siblings using upregulated genes identified in the microarray profiling of *rnaset2* ^*AO127*^. Expression analysis of immune response genes confirmed upregulation of *il1beta* and antiviral genes (interferon *ifnϕ1*, *mxa*, and *isg15*) (Figure 1d). Additionally, *rnaset2* expression was decreased threefold compared to WT siblings (Sup. Figure S6) suggesting nonsense‐mediated RNA decay. These results demonstrate that our new CRISPR/Cas9 *rnaset2* ^*sh532*^ mutant faithfully recapitulates the previous ENU‐generated mutant and can be used to further investigate the role of the immune system in the development of the disease.

To test whether the immune response is also upregulated in embryonic brain, we dissected heads of 5dpf larvae and used the same panel of genes used on 3‐month‐old brains (Figure 1e). The *il1beta* gene was found upregulated in 5dpf mutant brain suggesting the presence of an inflammatory response in embryonic brain. Interestingly, we found that this inflammatory response was not accompanied by an increased antiviral response as detected in older brain using the antiviral genes interferon *ifnϕ1*, *mxa*, and *isg15*. This points toward a potential mechanism involving an initial inflammatory stimulus during brain development, later followed by an antiviral response.

### 3.3 Embryonic brains of *rnaset2* ^*sh532*^ mutants contained profoundly abnormal microglia

**Figure 2:**
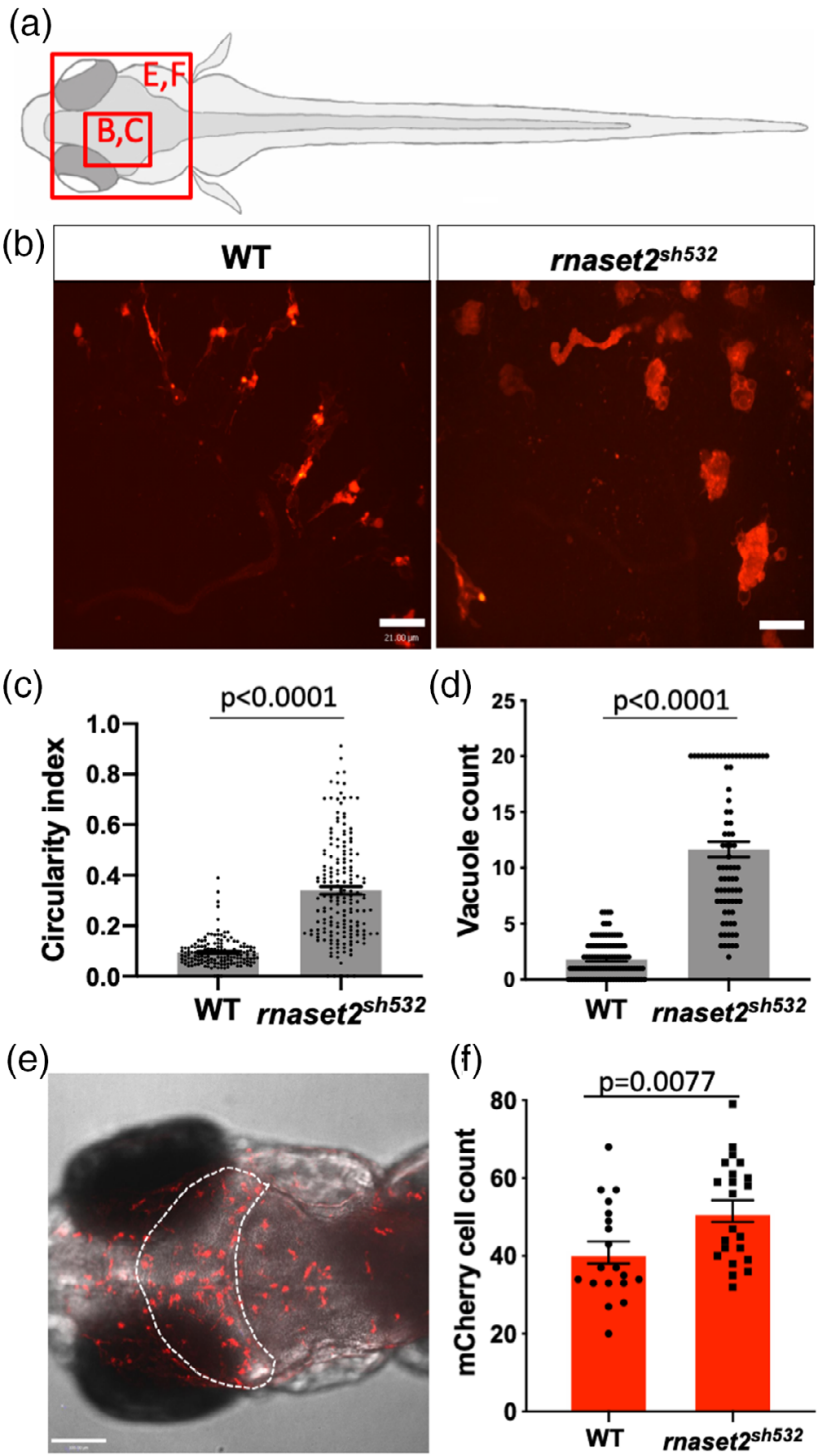
Microglia display impaired morphology and increased number in *rnaset2* ^*sh532*^ mutant (a) Diagram of a dorsal view of 5dpf zebrafish larvae with red boxes highlighting the brain region imaged in the panel (b) and (e). (b–d) Analysis of microglia circularity in the optic tectum in 5dpf wild type (WT) siblings and *rnaset2* ^*sh532*^ larvae. (b) Representative images of microglia morphology using the 40× objective. Scale bar 21 μm. (c) Quantification of microglia circularity using the circularity index analysis from Fiji. *n* = 48–50 larvae from three independent experiments, two‐tailed Mann–Whitney *U* test *p* = .6944. (d) Quantification of numbers of vacuole in individual microglia. Mutant counts were capped at 20 due to the lack of accuracy to identify vacuole when there were too many in each cell. *n* = 80–104 larvae from three independent experiments, two‐tailed Mann–Whitney *U* test *p* < .0001. (e,f) Microglia/macrophage count in the optic tectum (OT) of 5dpf WT siblings and *rnaset2* ^*sh532*^ larvae. Representative images of the whole head with OT outlined (dotted white line) (e) and quantification of mCherry positive cells from the *Tg*(*mpeg1*:*mCherryCAAX*)*sh378* reporter line labeling both microglia and macrophages (f). *n* = 19–22 larvae from three independent experiments, two‐tailed Mann–Whitney *U* test *p* = .0077 [Color figure can be viewed at wileyonlinelibrary.com]

To further characterize this inflammatory signal in the larval brain, we took advantage of the transparency of the zebrafish larvae to study the behavior of brain immune cells in vivo. Macrophages enter the brain at 2dpf and subsequently differentiate into microglia which are both labeled by the membrane reporter line *Tg*(*mpeg1*:*mCherryCAAX*)*sh378* (Ellett et al., 2011; Herbomel, Thisse, & Thisse, 2001). Confocal imaging of the optic tectum at 5dpf revealed profound defects in mutant microglia. Mutant microglia displayed an obvious abnormal phenotype, with a higher circularity index (Figure 2a–c, *p* < .0001) and containing a higher number of vacuoles labeled using the membrane transgenic line *Tg*(*mpeg1*:*mCherryCAAX*)*sh378* (Figure 2d, *p* < .0001). Interestingly, this engorged phenotype was reduced in homozygous mutants generated from a heterozygous in‐cross, suggesting that maternal contribution of *rnaset2* in early development is critical for maintaining a normal microglia morphology (Sup. Figure S7).

Increased immune response combined with abnormal microglia can potentially trigger a stress signal to recruit more immune cells in the brain. We counted the number of macrophages using the transgenic line *Tg*(*mpeg1*:*mCherryCAAX*)*sh378* and found a higher number of mCherry positive cells in mutant (Figure 2e,f, *p* = .0077). This result suggested that circulating macrophages infiltrated the brain.

### 3.4 Increased numbers of apoptotic cells in 5dpf *rnaset2* ^*sh532*^ mutants are due to defects in apoptotic cell degradation by microglia

**Figure 3:**
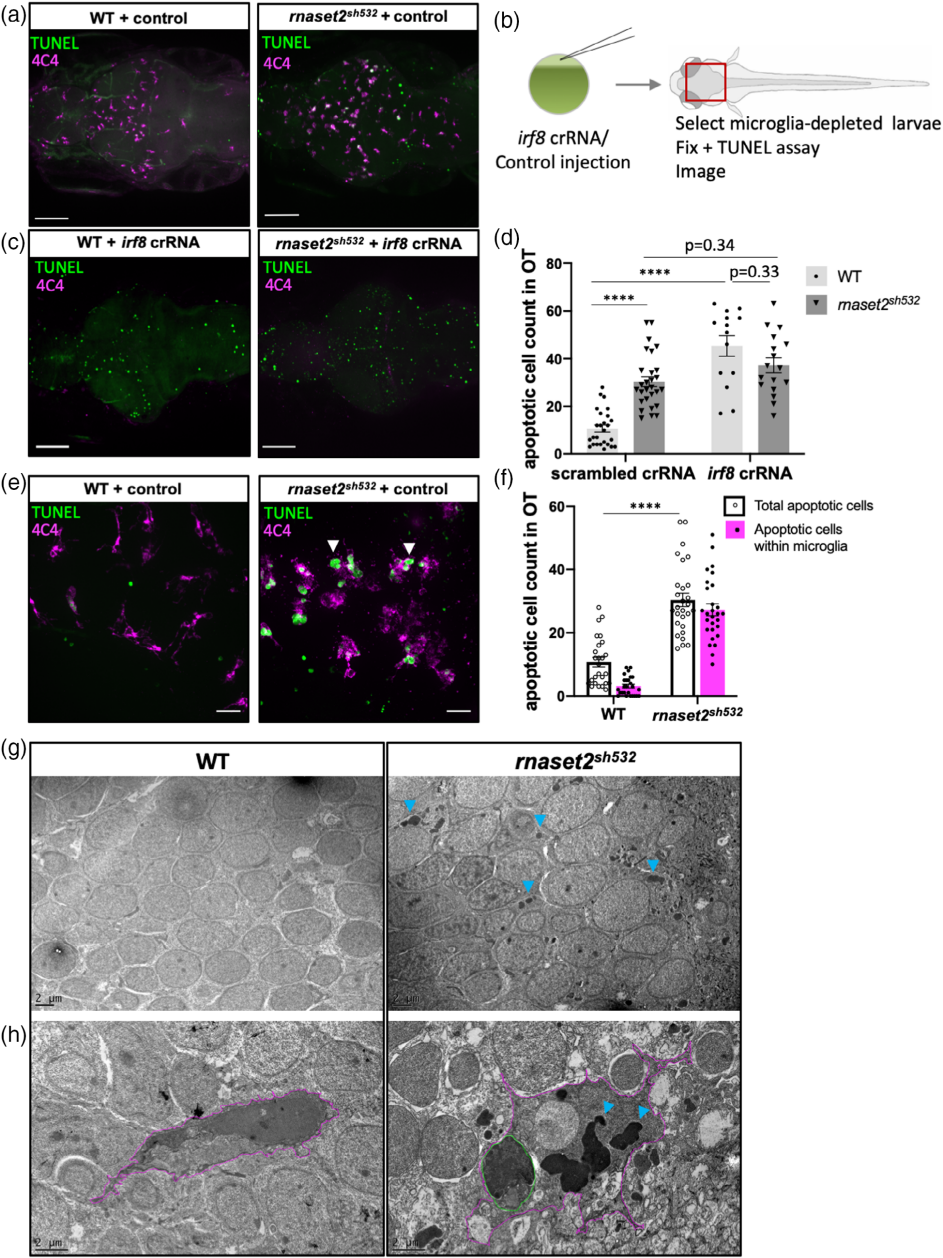
Increased number of apoptotic cells in 5dpf *rnaset2* ^*sh532*^ mutant is due to dysfunctional microglia. (a–d) Higher number of apoptotic cells in mutant is due to defective microglia and not excessive neuronal cell death. Representative images of apoptotic cells (as visualized by TUNEL staining) in brains of 5dpf *rnaset2* ^*sh532*^ mutants and wild type (WT) siblings in scrambled crRNA injected control (a) and following injection of *irf8* crRNA (c). Scale bar 70 μm. (b) Diagram of experimental flow. Injection of *irf8* crRNA or scrambled crRNA control was performed into one‐cell stage embryos, which were raised until 5dpf and microglia‐depleted larvae were selected in *irf8* crRNA injected animals. Control and depleted larvae were fixed and stained for apoptotic cells using TUNEL assay and microglia using 4C4 antibody. (d) Quantification of apoptotic cells in the optic tectum (OT). *n* = 15–28 from three independent experiments, using multiple two‐tailed *U* test with Bonferroni's multiple comparisons test (****p < .0001). (e–h) Undigested apoptotic cells are contained within microglia. (e) High resolution images of apoptotic cells (fluorescein TUNEL staining) and microglia (4C4 magenta) in brains of 5dpf *rnaset2* ^*sh532*^ mutants and WT siblings with apoptotic cells mainly appearing within microglia (white arrowheads). Scale bar 20 μm. (f) Quantification of total number of apoptotic cells and number of apoptotic cells contained within microglia. *n* = 26–28 from three independent experiments, using multiple two‐tailed *U* test with Bonferroni's multiple comparisons test (****p < .0001). (g,h) High‐resolution electron microscopy images of 5dpf *rnaset2* ^*sh532*^ mutants and WT siblings neurons (g) and microglia (h‐magenta) with dark inclusion bodies (blue arrowheads) and intact apoptotic cells (green) [Color figure can be viewed at wileyonlinelibrary.com]

Multiple signals can trigger macrophage brain entry, including neurodevelopmental apoptosis which was described as the first event to attract circulating macrophages into the developing brain (Casano, Albert, & Peri, 2016; Xu, Wang, Wu, Jin, & Wen, 2016). In zebrafish, this wave of neuronal apoptosis occurs between 1 and 2dpf and is accompanied by an influx of embryonic macrophages which differentiate into microglia and begin to phagocyte apoptotic cells—a clearance that is completed by 5dpf (Casano et al., 2016; Herbomel et al., 2001; Xu et al., 2016). To measure apoptosis in mutant brains, we labeled apoptotic cells with fluorescein using the TUNEL ApopTag kit. Quantification of apoptotic cell number in the optic tectum identified a more than twofold increase in mutant brains at 5dpf (Figure 3a,d, *p* < .0001). Interestingly, we found no difference at the earlier timepoint of 3dpf (Sup. Figure S8), suggesting that the initial number of dying neurons was unchanged in mutants.

We then hypothesized that the increased number of apoptotic cells observed at 5dpf was due the abnormalities identified in microglia. To test this hypothesis, we undertook a microglia‐depletion approach by targeting the transcription factor *irf8*, commonly used the block the production of embryonic macrophages (Li et al., 2011; Shiau, Kaufman, Meireles, & Talbot, 2015). Using a CRISPR/Cas9 approach with a single *irf8* crRNA resulted in a more robust depletion of microglia at 5dpf compared to the commonly used *irf8* antisense morpholino (Sup. Figure S9). As expected and previously shown (Villani et al., 2019), depletion of microglia in WT brains resulted in a threefold increase numbers of apoptotic cells compared to scrambled crRNA injected larvae at 5dpf (Figure 3b,–d, *p* < .0001). We found that depletion of microglia in *rnaset2* ^*sh532*^ mutants resulted in the same number of apoptotic cells versus control (Figure 3b–d, *p* = .34), suggesting that mutant microglia fail at clearing the brain from apoptotic cells even when present. Interestingly, depletion of microglia in mutant resulted in the same number of apoptotic cells than in depleted WT (Figure 3b–d). We therefore concluded that the rate of neuronal death during neurodevelopmental apoptosis is normal in mutant larvae. Furthermore, our results suggest that the increased number of apoptotic cells in *rnaset2* ^*sh532*^ mutants is due to a defect in apoptotic cell clearance by abnormal mutant microglia.

Clearing of apoptotic cells in the brain is performed by microglial phagocytosis, a process starting by the engulfment of dead cells and followed by degradation of the internalized cargo through fusion with lysosomes. We noticed that most apoptotic cells in mutant brains are in fact contained within microglia (Figure 3e,f), suggesting a defect in digesting, rather than ingesting, apoptotic cells. To confirm that mutant microglia can efficiently uptake dying neurons, we investigated the engulfment activity of mutant microglia during the clearing of neurodevelopmental apoptosis at 3dpf using live imaging. We injected the destination vector *ubiq*:*secAnnexinV‐mVenus* with Tol2 mRNA into the fluorescent reporter line *Tg*(*mpeg1*:*mCherryCAAX*)*sh378* to label apoptotic cells engulfment by microglia. Time‐lapse videos showed that mutant microglia were able to migrate toward dying cells, form a phagocytic cup and phagocytose dying cells at 3dpf (Sup. Figure S10c, Video S1). The number of engulfed apoptotic cells was quantified on still images and showed no difference between mutant and WT microglia (Sup. Figure S10b).

To understand how microglia could fail at digesting apoptotic cells, we used EM to obtain high‐resolution images of microglial intracellular structure. Images of neurons from 5dpf mutant samples confirmed that most of the neurons contained large electron dense inclusions within their cell bodies as already identified in the previous ENU *rnaset2* ^*AO127*^ mutant (Figure 3g) (Haud et al., 2011). EM images of mutant microglia showed enormous electron dense inclusions and intact apoptotic cells contained within the microglia cell body (Figure 3h). We therefore concluded that the higher count of apoptotic cells in the brains of *rnaset2* ^*sh532*^ mutants is not due to impaired microglial engulfment but a defect in digesting their content. This is reminiscent of a defect in the lysosomal degradation pathway and reinforces our initial published proposal that loss of *rnaset2* causes an LSD (Haud et al., 2011).

### 3.5 Microglial‐specific rescue of *rnaset2* restores embryonic phenotype in *rnaset2* ^*sh532*^ mutant

**Figure 4:**
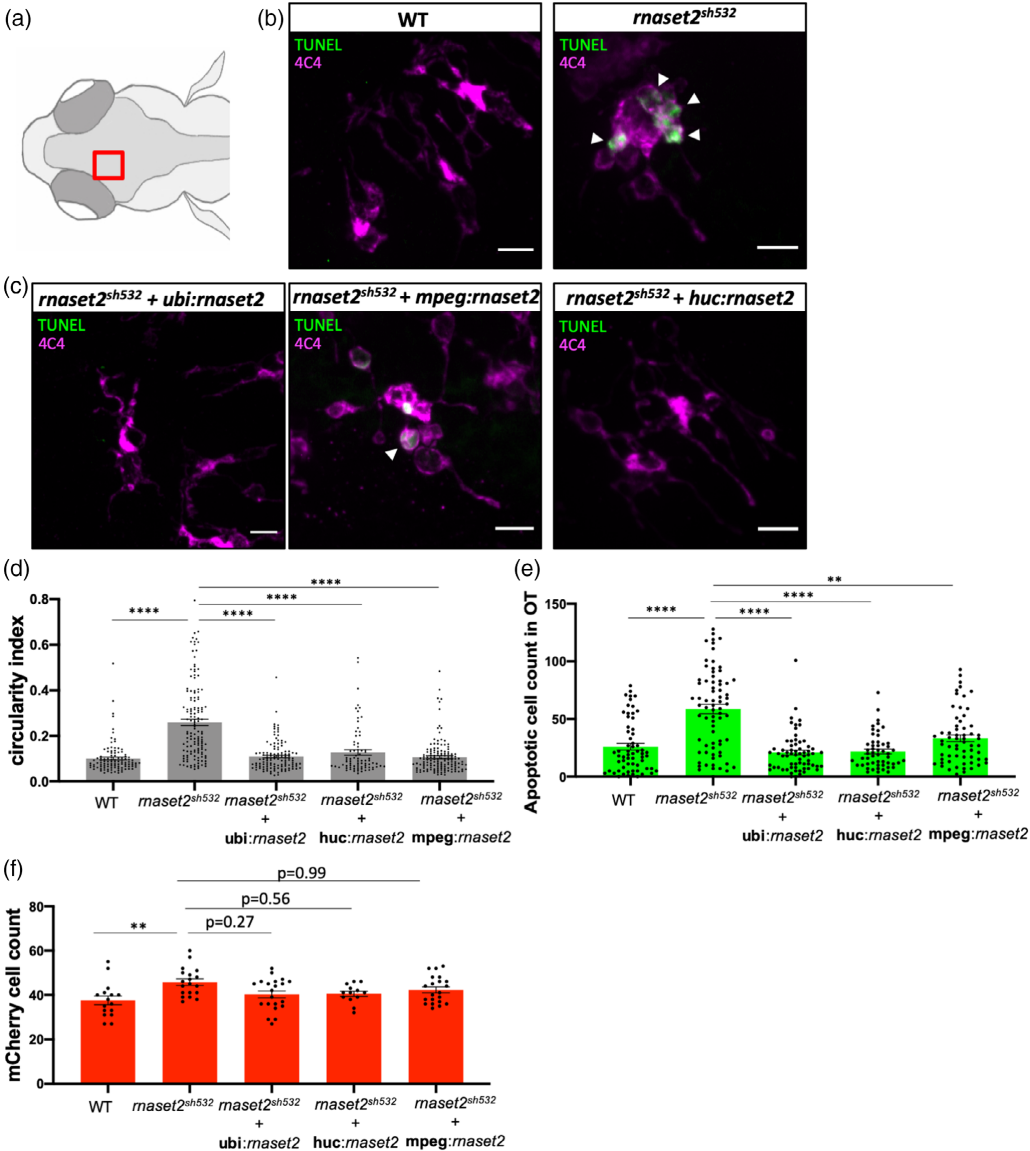
Tissue‐specific rescue of *rnaset2* restores embryonic phenotype in *rnaset2* ^*sh532*^ mutant. (a) Diagram of a dorsal view of a 5dpf zebrafish head with red box highlighting the region imaged in the next panels. (b) Representative confocal images of apoptotic staining (TUNEL) combined with immunofluorescence for 4C4 labeling microglia in brains of 5dpf wild type (WT) siblings and *rnaset2* ^*sh532*^ larvae. Scale bar 3 μm. (c) Representative confocal images of apoptotic staining (TUNEL) combined with immunofluorescence for 4C4 labeling microglia in stable F1 5dpf *rnaset2* ^*sh532*^ larvae from founders injected at one‐cell stage to rescue expression of the *rnaset2* gene either ubiquitously (*ubi*), or in macrophages/microglia (*mpeg*), or in neurons (*huc*). Scale bar 3 μm. (d) Quantification of microglia circularity measuring the circularity index. *n* = 78–141 microglia from three independent experiments, Kruskal–Wallis test with Dunn's multiple comparisons *p* < .0001. (e) Quantification of number of apoptotic cells by TUNEL staining. *n* = 53–72 larvae from three independent experiments, Kruskal–Wallis test with Dunn's multiple comparisons *p* < .0001. (f) Macrophage/microglia mCherry count after anti‐RFP immunofluorescence staining. *n* = 16–21 larvae from three independent experiments, Kruskal–Wallis test with Dunn's multiple comparisons *p* = .0152 [Color figure can be viewed at wileyonlinelibrary.com]

Our findings suggest a new model whereby lysosomal defects prevented microglia to digest apoptotic neurons during neurodevelopmental apoptosis. As such, this LSD mechanism suggests that replacing functioning rnaset2 protein within microglia will present a therapeutic strategy to rescue the embryonic phenotype. There are two possible approaches to restore functional rnaset2 in microglia: directly by targeting microglia, or indirectly through neuronal rescue. Neuronal rescue would resemble a gene therapy‐like approach, allowing the missing lysosomal *rnaset2* protein to reach surrounding microglia through exocytosis (cross correction) or phagocytosis of dying neurons. We designed a genetic tissue‐specific rescue approach to restore *rnaset2* expression in different cell types, using the *ubi* promoter for ubiquitous expression serving as positive control, the *mpeg* promoter to specifically target the macrophage and microglia linage (Ellett et al., 2011), and the *huc* promoter to target neurons. Rescue plasmids were built using gateway cloning and all constructs contained a WT *rnaset2* middle entry vector, and a blue eye marker to allow screening of positive larvae. To avoid mosaicism and ensure a stable expression of the *rnaset2* gene, F0 injected blue‐eyed larvae were raised and screened for founders. F1 blue‐eyed larvae from separate founders were fixed at 5dpf and subsequently analyzed for microglial and apoptotic cell number analysis. As expected, the positive control *ubi*:*rnaset2* vector rescued the brain phenotype observed in the *rnaset2* ^*sh532*^ mutants, such that there was no difference in microglia circularity index compared to WT microglia (Figure 4a–d, *p* < .0001), and no different in number of apoptotic cells (Figure 4a–c,e). Reexpression of *rnaset2* specifically in macrophages and microglia also rescued microglial morphology and numbers of apoptotic cells (Figure 4a–e, *p* < .001) and interestingly the same was observed with the neuronal rescue (Figure 4a–e, *p* < .0001). Microglia cell count was partially rescued by all constructs, although this did not reach statistical significance (Figure 4f, *p* = .0152). These results demonstrated that targeting microglia either directly or indirectly can restore microglial morphological defects and their ability to digest apoptotic neurons. This highlights cell‐specific strategies for targeted therapy, representing exciting therapeutic avenues for treatment of *rnaset2* ^*sh532*^ deficient leukoencephalopathy.

### 3.6 *rnaset2* ^*sh532*^ mutants exhibit decreased survival and abnormal behavior in adult and larval stages

**Figure 5:**
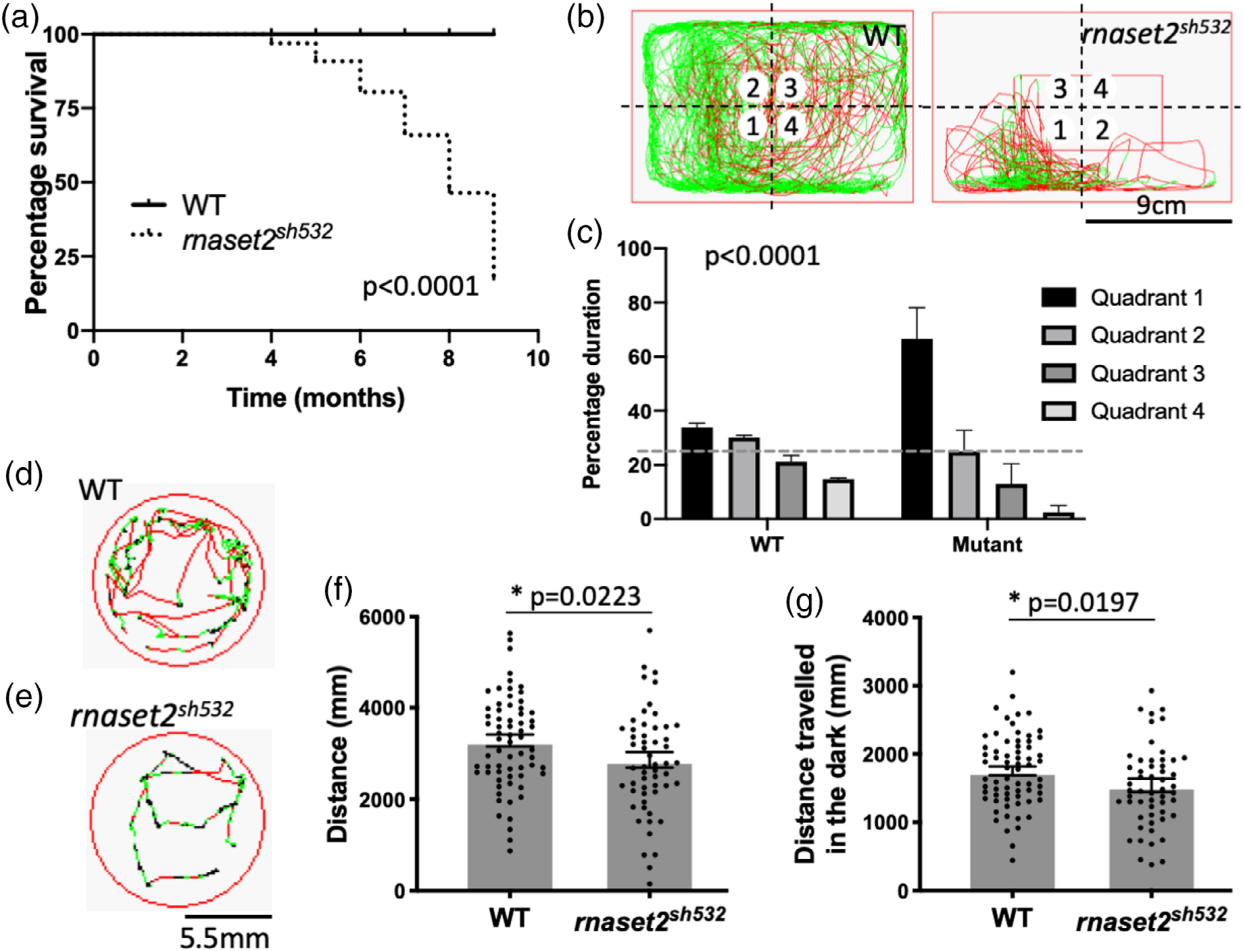
*rnaset2* ^*sh532*^ mutants exhibit decreased survival and abnormal behavior in adult and larval stages. (a) Survival curve of *rnaset2* ^*sh532*^ and wild type (WT) sibling. Death outcome is represented by humanely culling animals displaying a spiraling swimming phenotype. Log rank Mantel–Cox test, *p* < .0001. *n* = 22. (b) Representative traces of movement from 8‐month‐old adults WT siblings and *rnaset2* ^*sh532*^ in all four quadrants, where quadrant number 1 representing the quadrant most visited and Quadrant 4 the least visited. Scale bar representing the size of the tank used for the test. (c) Quantification of quadrant analysis in adult. *n* = 4 using a contingency table and a Chi‐square analysis *p* < .0001. (d,e) Examples of movement from 5dpf WT siblings (d) and *rnaset2* ^*sh532*^ larvae during 1 min (e). (f,g) Swimming activity of 5dpf embryos as measured by distance traveled across a 20 min period of alternating light and dark (f) and in dark phases only (g). *n* = 56–67 larvae from three independent experiments, two‐tailed Mann–Whitney *U* test *p* = .0223 (f) and *p* = .0482 (g) [Color figure can be viewed at wileyonlinelibrary.com]

As the first generation of homozygous and WT siblings generated from genotyped F2 reached adulthood, we noticed a clear tilted swimming phenotype in 8‐month‐old *rnaset2* ^*sh532*^ mutants (Sup. Video S2). Tilted swimming behavior deteriorated into abnormal spiraling (Sup. Video S3), at which point they were not able to feed properly and were humanely culled. For this reason, most of *rnaset2* ^*sh532*^ mutants had to be culled before reaching 12 month old while WT siblings did not develop a tilted or spiraling swimming behavior at any stage (Figure 5a). To perform an unbiased swimming behavior analysis on the rest of the clutch, we used the ViewPoint system and recorded normal swimming behavior for 10 min. WT adults exhibited exploratory behaviors, demonstrated by visiting all four quadrants of the tank equally (Figure 5b,c). Swimming and exploratory behaviors were significantly altered in *rnaset2* ^*sh532*^ adults, which swam in repetitive stereotyped patterns restricted to a single quadrant in which they spent the majority of their time (Figure 5b,c). Interestingly, swimming speed and distance traveled was not different (Sup. Figure S11).

RNAseT2‐deficient patients present with severe locomotor disabilities, including spasticity and dystonia, which arise in the first year of the patient life (Henneke et al., 2009). To assess locomotion in early life, we performed locomotion tests at 5dpf using the Zebrabox ViewPoint system. We measured the swimming activity of 5dpf *rnaset2* ^*sh532*^ mutants relative to age‐matched WT siblings over 20 min, alternating light and dark phases (Pant, Boespflug‐Tanguy, & Pujol, 2019). We found that *rnaset2* ^*sh532*^ larvae were less active compared to WT siblings (Figure 5d–g). Total swimming distance over a period of 20 min of alternating light and dark was decreased in mutants compared to WTs, with hypoactivity accentuated in dark phases (Figure 5f,g, *p* = .0223, *p* = .0482). These early onsets of locomotor abnormalities and increased immune response in the *rnaset2* ^*sh532*^ larvae suggest that the brain integrity might be compromised during early stages of development.

## 4 DISCUSSION

Using the transparency and the ex utero development of the zebrafish embryos, we were able to identify profound microglia abnormalities during neurodevelopment for the first time in a model of human leukodystrophy. This study identified dysfunctional microglia as a new early marker of the pathology in RNAseT2‐deficient leukoencephalopathy. Our results also demonstrate that microglia‐specific interventions can rescue microglial defects in *rnaset2*‐deficient zebrafish, therefore highlighting a cell specific therapeutic approach to be investigated in patients.

RNAseT2‐deficient leukoencephalopathy was already proposed to be classified as an LSD from our previous study, showing accumulation of rRNA in neurons (Haud et al., 2011). In our new CRISPR/Cas9 model, we showed that mutant microglia can detect and uptake apoptotic cells normally during neurodevelopmental apoptosis but failed to break down their ingested cargo. This resulted in a higher number of apoptotic cells detected in mutant, mainly contained within mutant microglia. Electron microscopy confirmed our previously described dark inclusions in mutant neurons (Haud et al., 2011) and identified much larger inclusions in microglia alongside intact apoptotic cells. This is indeed reminiscent of an LSD and reinforces our initial proposal. The specific “engorged” morphology and the accumulation of undigested substrate identified the microglia as the bottleneck of the system. Undigested substrates can affect the cell in multiple ways, including impaired degradation of pathogens by macrophages, disrupted lysosome positioning in the cell or autophagy build‐up (Marques & Saftig, 2019). Here, we showed that accumulation of apoptotic cells in saturated microglia could be the start of the pathology. Although these microglial defects have not yet been described in RNAseT2‐deficient leukoencephalopathy patients, they still represent an early marker of the pathology and a potential therapeutic target for RNASET2‐deficient leukoencephalopathy.

To our knowledge, this is the first time that neurodevelopmental apoptosis has been linked to defective microglial function in a LSD. Nearly 50% of neurons die during neurodevelopmental apoptosis (Dekkers et al., 2013; Nijhawan et al., 2000)—a key step of brain development that relies on microglia to clear away dead cells. Like many other LSDs and leukodystrophies, RNASET2‐deficient leukoencephalopathy affects patients during the early years of life, suggesting a fetal initial insult (Henneke et al., 2009; Rutherford & Hamilton, 2019). Developmental apoptosis represents the first scavenging challenge for microglia, and although considered a normal physiological event, we showed here that this extreme number of apoptotic cells to clear could have a profound impact on microglia.

Increased apoptosis in the brain acts as a signal for brain entry of macrophages (Casano et al., 2016; Xu et al., 2016), which can explain the increased number of microglia/macrophages observed in mutant brains. Engorged microglia could potentially secrete stress factor, become neurotoxic and trigger cumulative arrival of macrophages. Our study identifies an early inflammatory signature in 5dpf larval brain, but future microglia‐specific transcriptomic studies will be informative to elucidate their inflammatory profile.

Tissue‐specific rescue of *rnaset2* expression using a neuronal or a macrophage/microglia promoter restored microglia morphology and the number of apoptotic cells present in mutant brains. Neuronal restoration of normal *rnaset2* expression could contribute to microglial rescue. Deficient microglia could inherit the corrected version of the protein by “cross‐correction,” a common phenomenon for lysosomal enzymes that are secreted into the extracellular space and captured by surrounding cells often observed after gene therapy or bone marrow transplant in LSD patients (Fratantoni, Hall, & Neufeld, 1968; Penati, Fumagalli, Calbi, Bernardo, & Aiuti, 2017; Rastall & Amalfitano, 2015; Walkley et al., 1994).

Most importantly, to our knowledge, our zebrafish model is the first de novo animal model of a leukodystrophy to recapitulate the patient neuropathology throughout development and into adulthood. Murine models of leukodystrophies have failed to reproduce all aspects of the human disease, with a recent RNASET2‐deficient rat model showing deficiencies in object recognition memory but no locomotor or spatial memory defects (Sinkevicius et al., 2018). *rnaset2* zebrafish mutants show hypoactivity in larval stages and tilted swimming in adulthood with an impaired exploratory phenotype. The adult tilted swimming phenotype can be caused by defects in the vestibular righting reflex or motor deficit (Kalueff et al., 2013). Such vestibular defects may be due to impaired ear development or impairment of sensory neurons within the inner ear (Whitfield, 2002) and may therefore recapitulate the sensorineural hearing loss diagnosed in patients (Henneke et al., 2009). Hence, our study places the *rnaset2* ^*sh532*^ zebrafish mutant at the forefront of leukodystrophy preclinical animal models and will be used to develop therapies targeting the microglial population.

Our study identifies an important role for the ribonuclease T2 protein in early brain development and reveals that deficient microglia could underpin the pathology of RNAseT2‐deficient leukoencephalopathy. We provide evidence to focus on therapies that can target the microglial population, such as gene therapy and hematopoietic stem cell transplantation. These therapies are already being used in clinical trials for LSDs and other types of leukodystrophies (Penati et al., 2017; Rutherford & Hamilton, 2019; Schiller, Henneke, & Gärtner, 2019) and could therefore be adapted to treat children suffering from RNAseT2‐deficient leukoencephalopathy.

## CONFLICT OF INTEREST

The authors declare no potential conflict of interest.

## Supporting information


Figure S1

Figure S2

Figure S3

Figure S4

Figure S5

Figure S6 S7

Figure S8

Figure S9

Figure S10

Figure S11

Table S1

Video S1

Video S2

Video S3

## Data Availability

The data that support the findings of this study are openly available in GEO at https://www.ncbi.nlm.nih.gov/geo/query/acc.cgi?acc=GSE138493, reference number GSE138493.
