# Supplementary figures and images for: The failure of microglia to digest developmental apoptotic cells contributes to the pathology of RNASET2‐deficient leukoencephalopathy
Source: Glia. 2020 Mar 25;68(7):1531–45. doi: 10.1002/glia.23829 (PMC8647916; doi:10.1002/glia.23829)

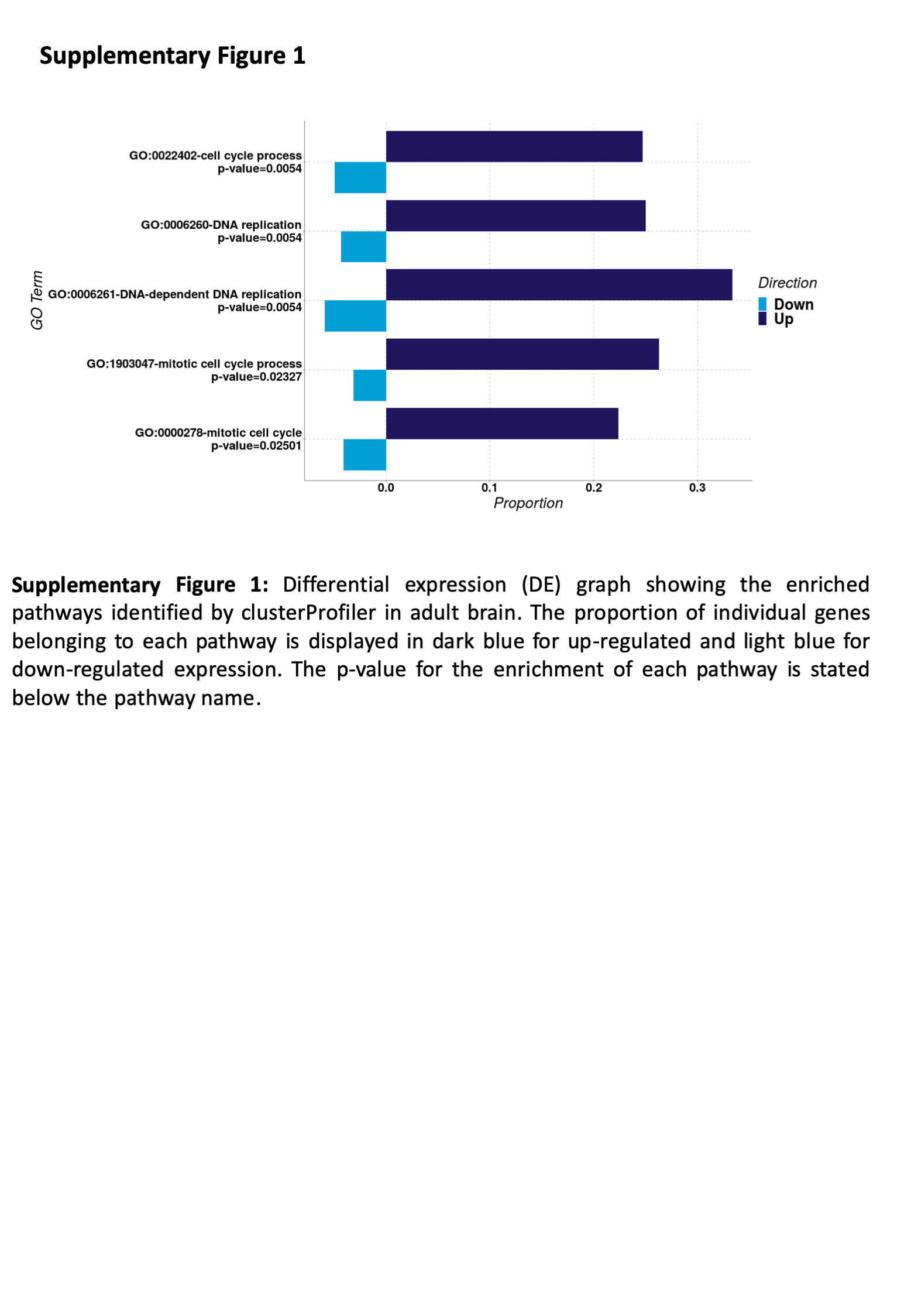

Supplement: Supplementary file 1 — Figure S1 [file GLIA-68-1531-s001.tif]

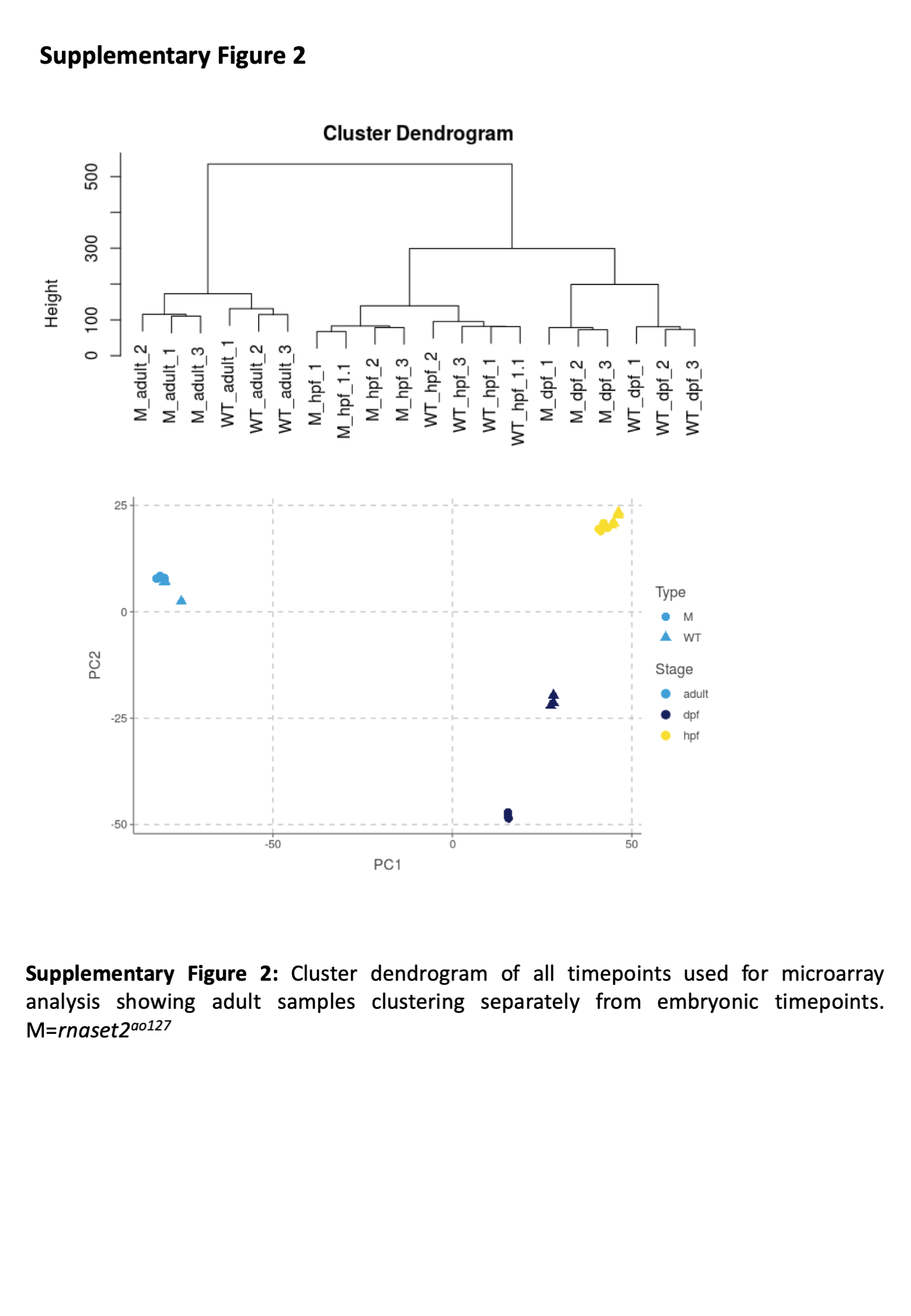

Supplement: Supplementary file 2 — Figure S2 [file GLIA-68-1531-s012.tif]

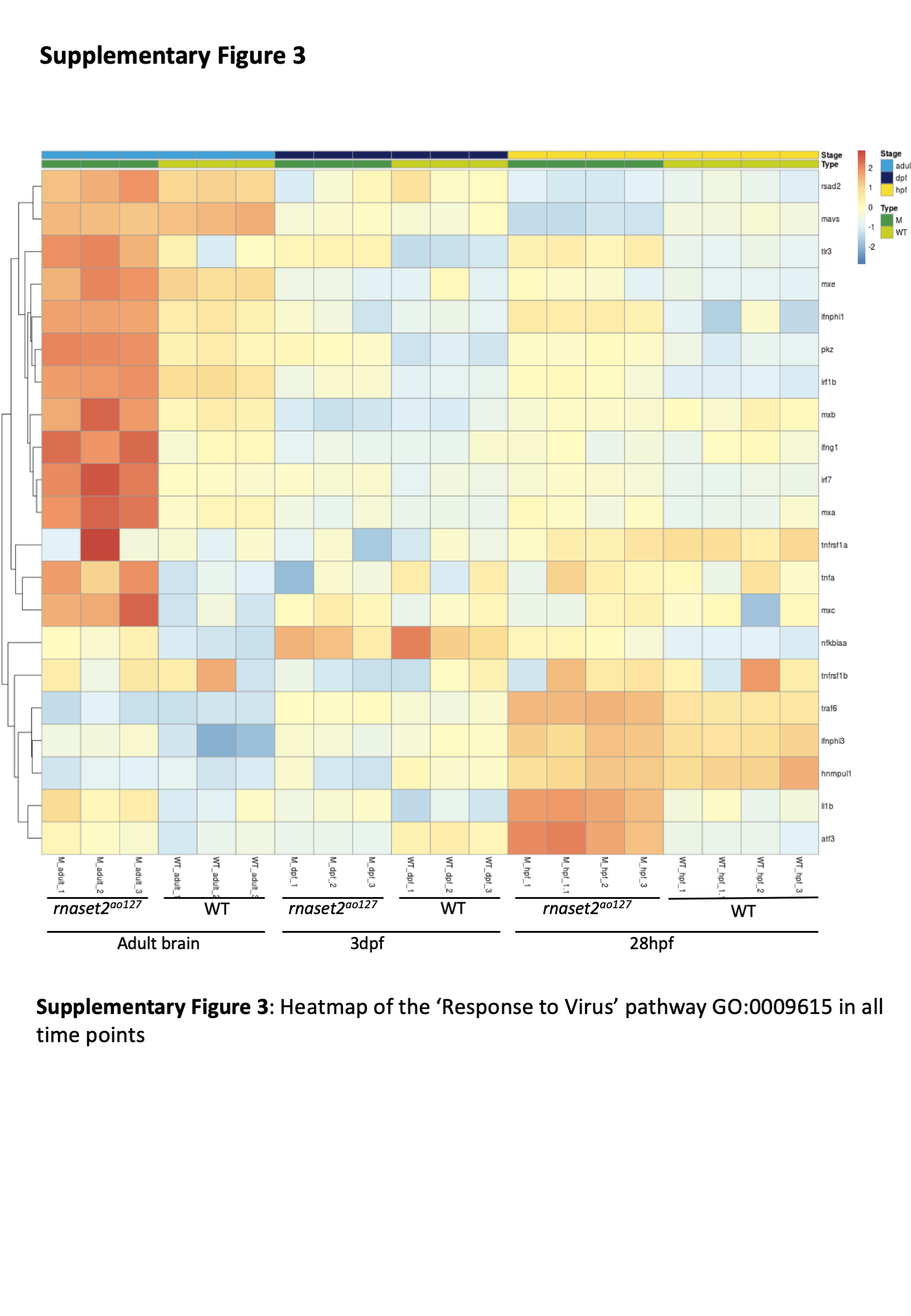

Supplement: Supplementary file 3 — Figure S3 [file GLIA-68-1531-s011.tif]

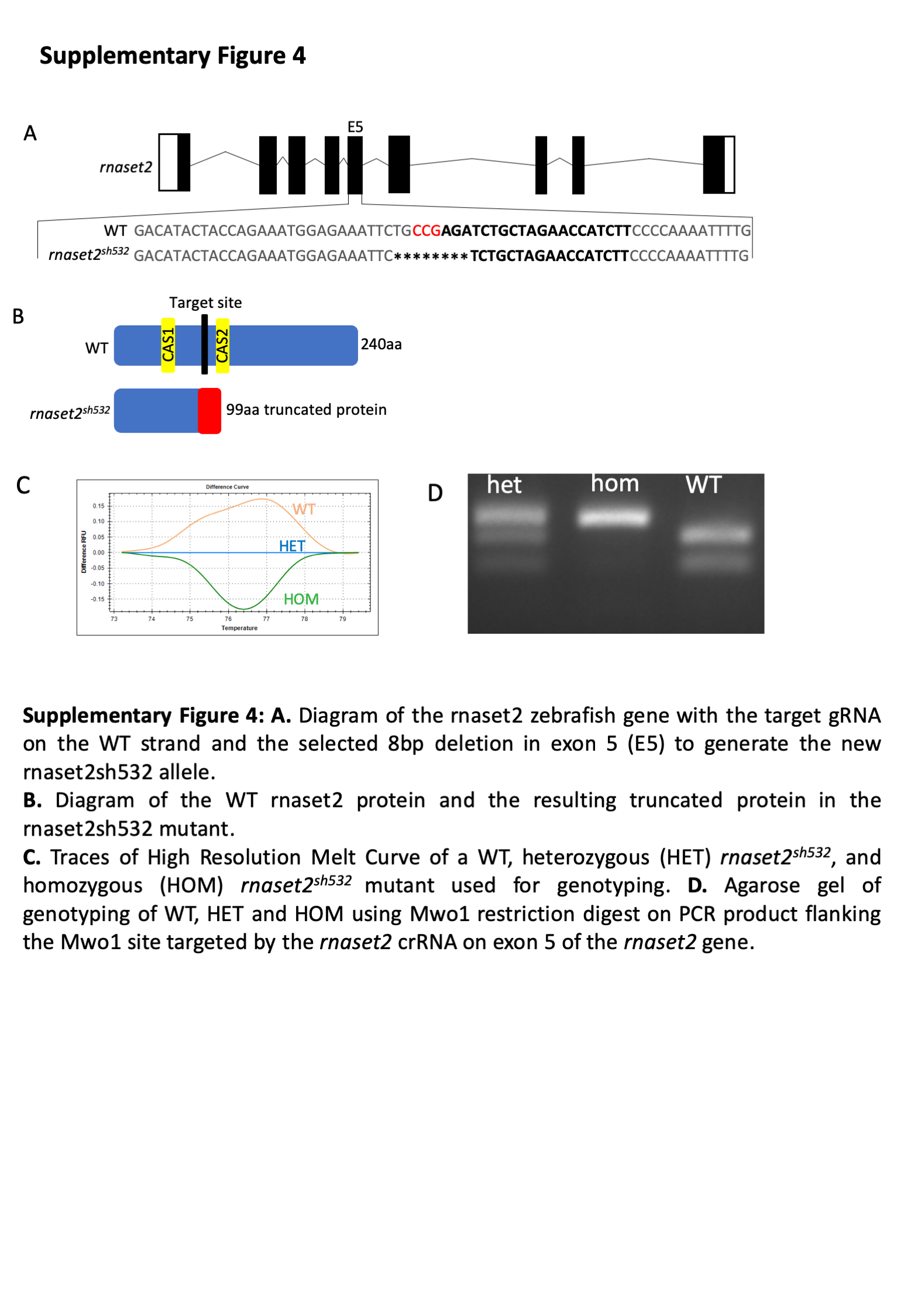

Supplement: Supplementary file 4 — Figure S4 [file GLIA-68-1531-s013.tif]

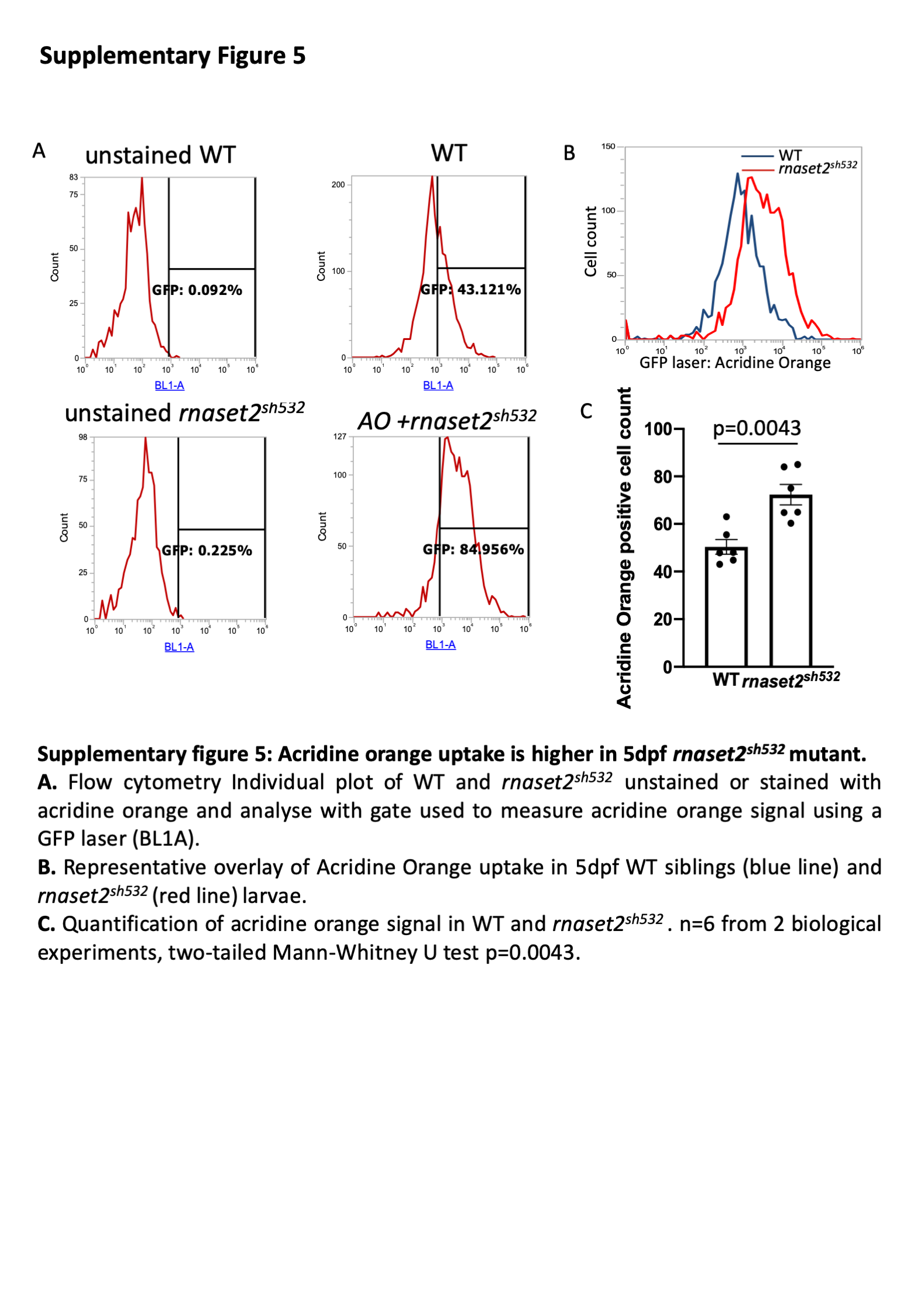

Supplement: Supplementary file 5 — Figure S5 [file GLIA-68-1531-s014.tif]

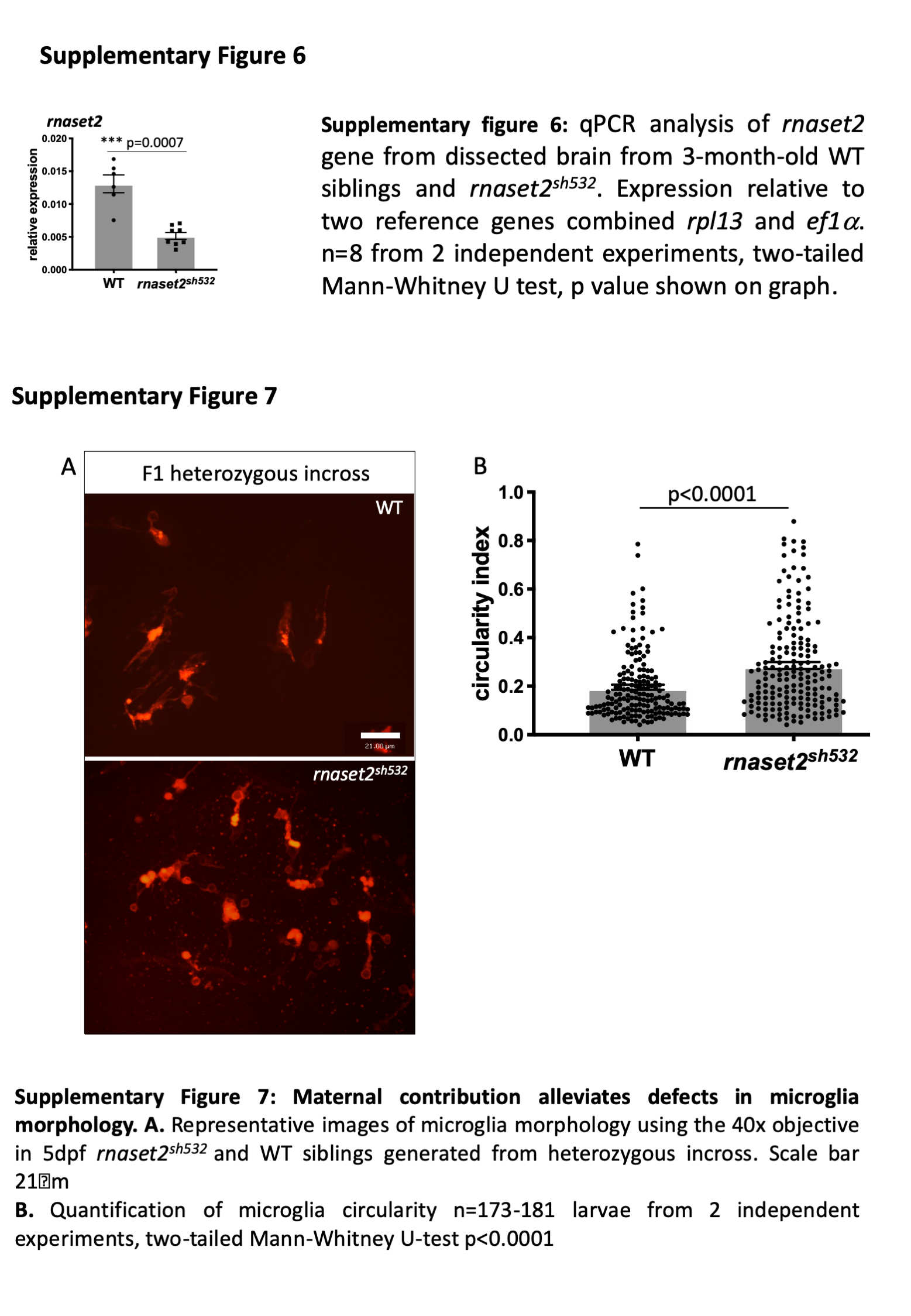

Supplement: Supplementary file 6 — Figure S6 S7 [file GLIA-68-1531-s002.tif]

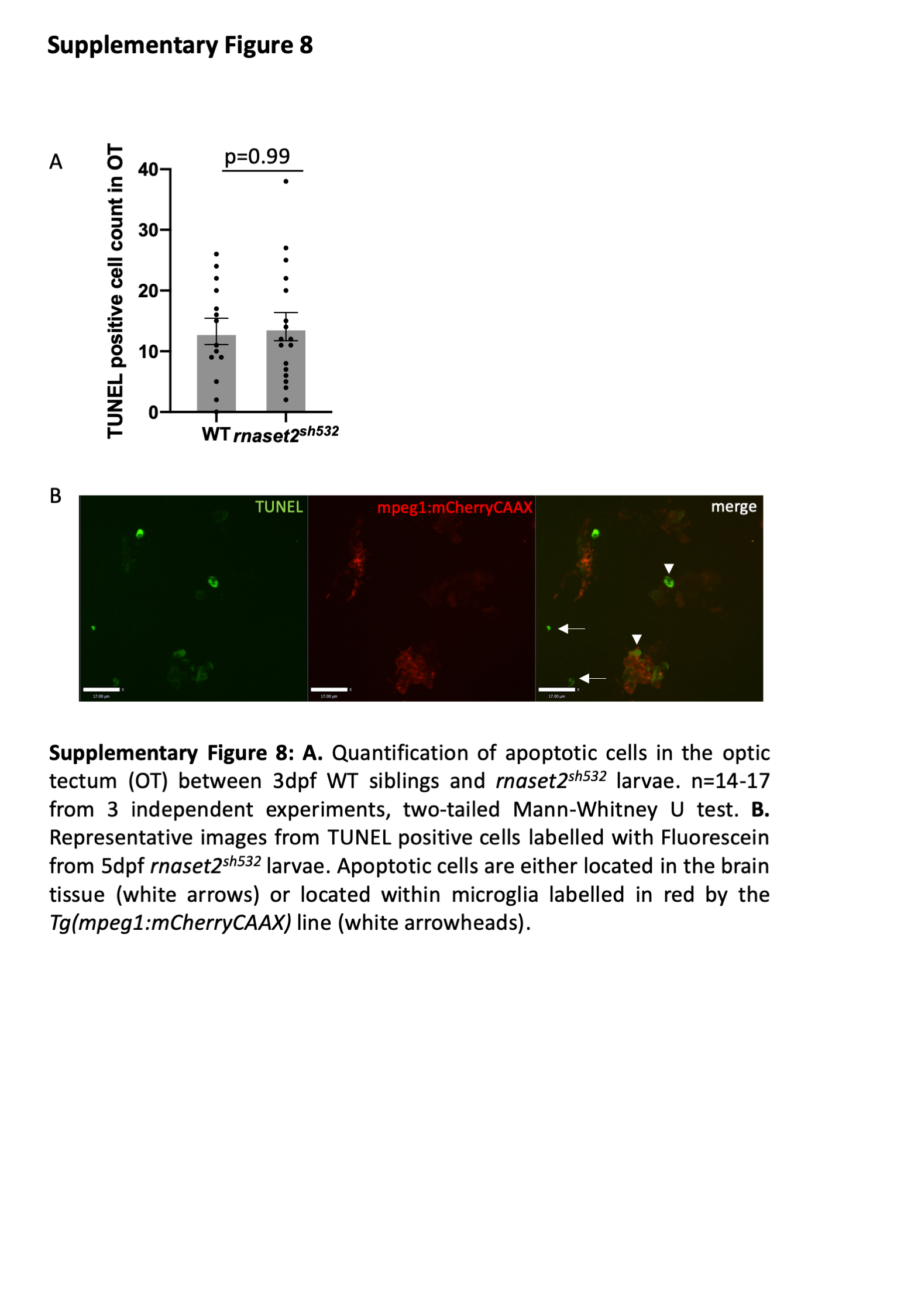

Supplement: Supplementary file 7 — Figure S8 [file GLIA-68-1531-s003.tif]

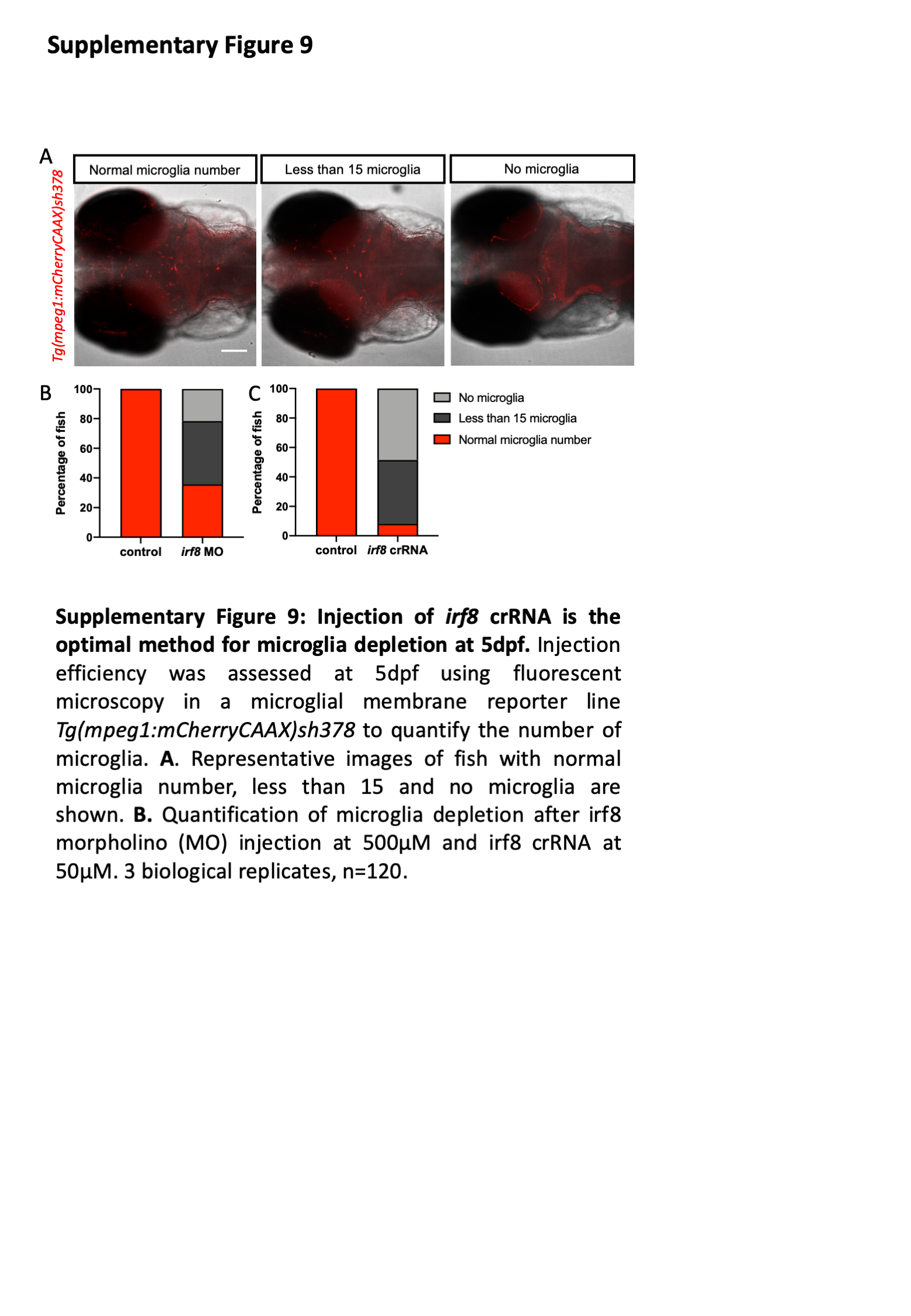

Supplement: Supplementary file 8 — Figure S9 [file GLIA-68-1531-s004.tif]

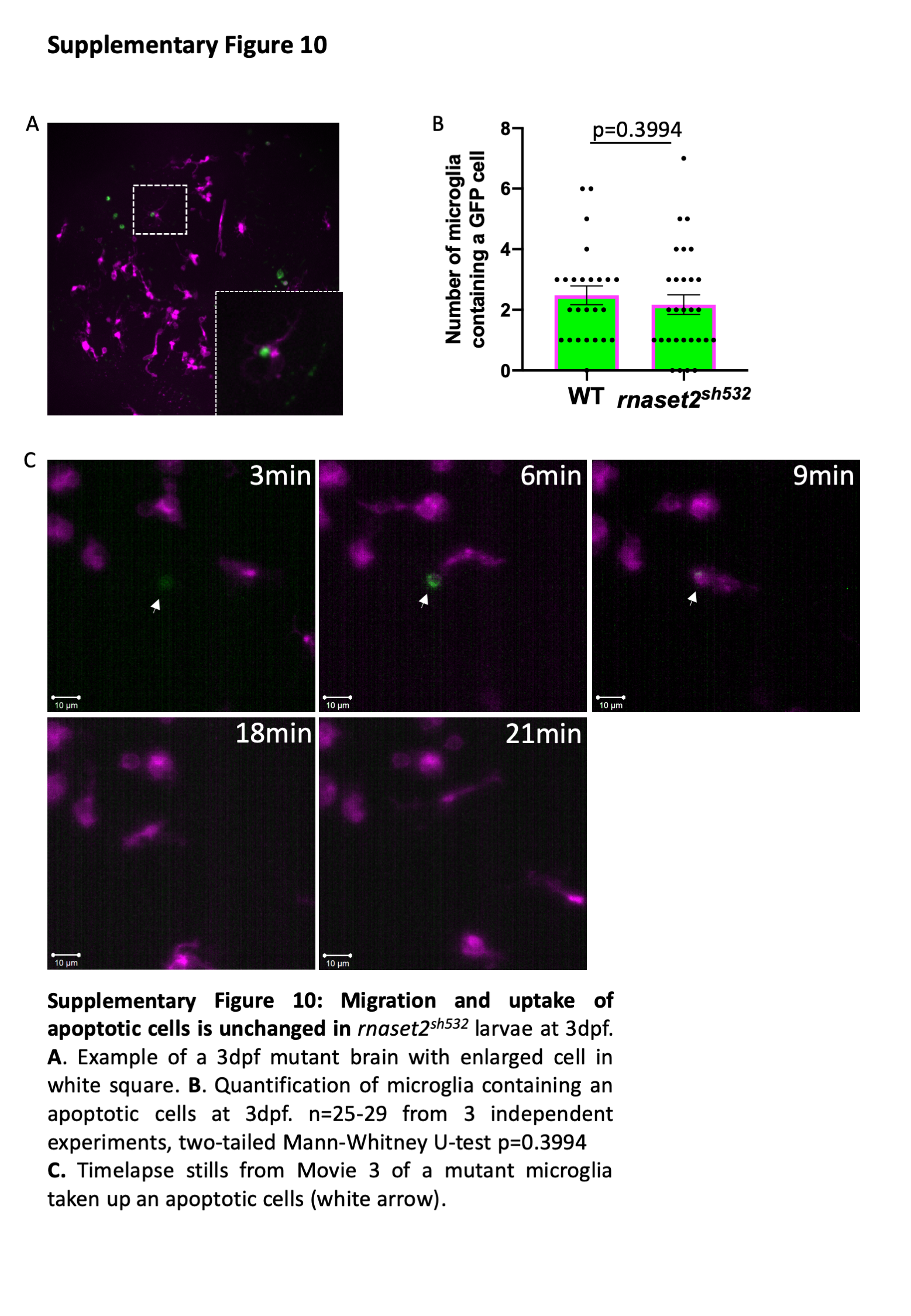

Supplement: Supplementary file 9 — Figure S10 [file GLIA-68-1531-s005.tif]

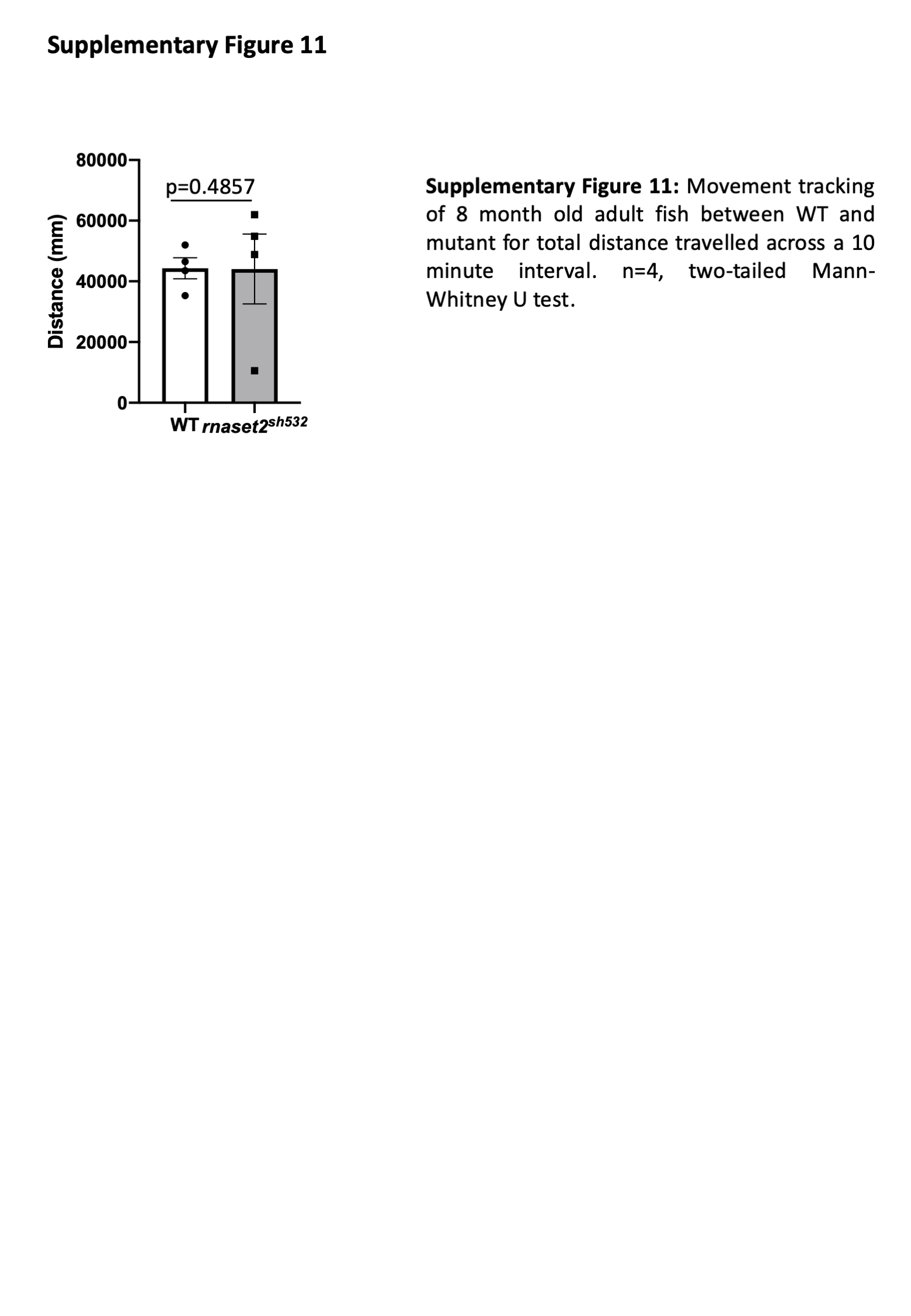

Supplement: Supplementary file 10 — Figure S11 [file GLIA-68-1531-s006.tif]

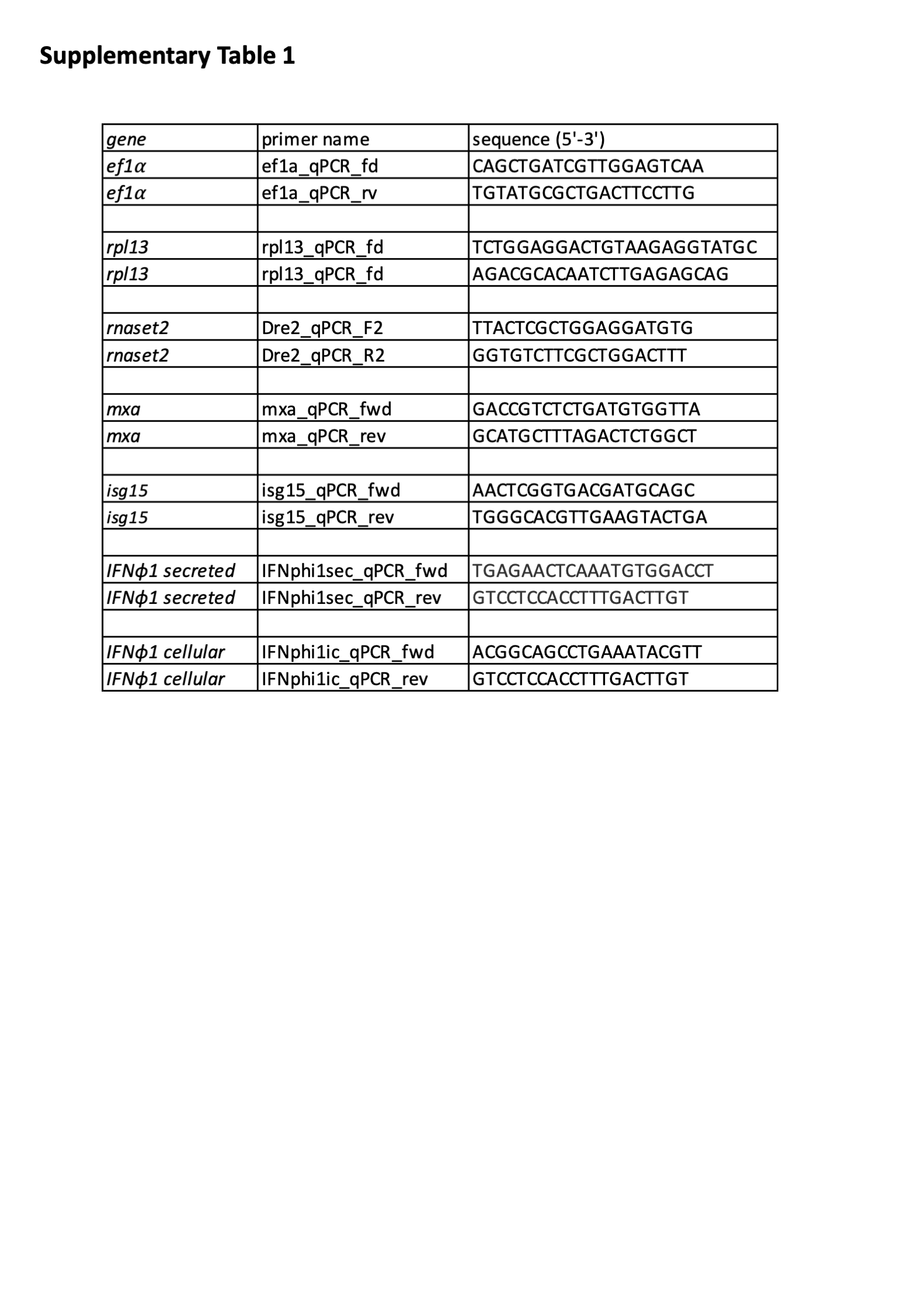

Supplement: Supplementary file 11 — Table S1 [file GLIA-68-1531-s007.tif]
